# When to Not Respond in Kind? Individuals’ Expectations of the Future and Their Support for Reciprocity in Foreign Policy

**DOI:** 10.1007/s11109-023-09857-y

**Published:** 2023-01-16

**Authors:** Osman Sabri Kiratli, Sabri Arhan Ertan

**Affiliations:** 1 International Trade Department, Bogazici University, Istanbul, Turkey; 2 WZB, Berlin, Germany

**Keywords:** International cooperation, Reciprocity, Public opinion, International institutions, Trade liberalization

## Abstract

This paper investigates if individuals’ negative assessments of the future drive micro-level reluctance for international cooperation and reciprocal behavior, a core principle of multilateralism. To test our theoretical expectations, we field online survey experiments on a sample of over 3000 respondents in the US and Turkey in October–November 2020. The experimental results show that on average, individuals are fairly sensitive to target countries’ policy actions and are inclined to reciprocate when contemplating whether to increase contributions to UN or consent to bilateral trade liberalization. Yet, further analyses concur that individual inclinations to reciprocate are substantially moderated by their future expectations. Specifically, individuals who are more pessimistic about their material prospects remain fairly indifferent to the positive actions of other countries, but are more likely to penalize negative foreign policy actions by reciprocating in kind.

**Supplementary Information:**

The online version contains supplementary material available at 10.1007/s11109-023-09857-y.

## Introduction

For many observers of global politics, the rule-bound system of post-war world order is under strain. A wave of political opposition against international organizations (IOs) and regimes had been gaining traction in the last decade (e.g., Bearce & Scott, 2019; Börzel & Zürn, 2021). The rising mass perception that many IOs have failed to perform and meet their founding objectives provides ample opportunities for leaders, particularly populists, to mobilize nationalist sentiments against multilateralism, resulting in new challenges against both the input and output legitimacy of cooperative frameworks. As De Vries et al. note (2020), this growing public mobilization against IOs reduces governments’ willingness to enter into new multilateral regimes and threatens their credibility when they do so.

A core operating principle of international cooperation is reciprocity, which refers to “exchanges of roughly equivalent values in which the actions of each party are contingent on the prior actions of the others in such a way that good is returned for good, and bad for bad” (Keohane, 1986, p. 8). Under reciprocity, actors reward the cooperative—and penalize the uncooperative—initiatives of the other party. As each actor assumes their actions will be reciprocated by the other party, they avoid uncooperative policies. Reciprocal behavior facilitates economic exchanges, helps sustain long-term transactions and consequently yields efficiency gains when incentives to cooperate are weak and enforcement mechanisms are absent (Buchan et al., 2006; Fehr et al., 1997).

This paper aims to explore individuals’ inclination to reciprocate in two domains of international cooperation: contributions to international organizations and bilateral trade relations. Theoretically, we hypothesize that, on average, citizens are sensitive to the actions of other countries when evaluating proposals on trade and cooperation through IOs. Yet, this general tendency to reciprocate becomes more nuanced when linked to their future economic assessments. We argue that when citizens expect intensified resource competition and shortened shadow of the future, they would prefer immediate—but lower—payoffs gained by defecting at the expense of long-term—though potentially higher—benefits from reciprocal cooperation. Consequently, we propose, the more pessimist individuals are with regards to their material well-being, the less likely they are to value reciprocal behavior at the international level. Though these pessimist individuals will still reciprocate against the uncooperative foreign policy actions of other countries, they will react to cooperative policy actions either by not responding in kind or by countering with a hostile response.

The empirical analysis is based on data gathered from an online survey with a novel experimental module conducted in late 2020 on samples of over 1500 adult respondents each in the US and Turkey. The experimental design adopts two factorial vignettes in which respondents are presented with independent short scenarios that describe several countries’ decisions to possibly change their contributions to the UN and their tariff rates vis-à-vis survey country exports, respectively. After each scenario, respondents are questioned on their preferences for their country’s foreign policy actions with respect to these two policy domains.

Our results show that in general, individuals in both samples are fairly sensitive to other countries’ policy actions and are keen to reciprocate in their policy preferences. However, these two country samples differ substantially on how they respond to positive and negative signals by other countries. In the US, the survey respondents reward cooperative behavior more than they punish uncooperative behavior, whereas the reverse is the case in Turkey. Further analyses concur that though individuals’ material worries do not necessarily reduce their support for cooperative frameworks, they substantially modify respondents’ reciprocal motivations. Specifically, individuals who are pessimistic about their future economic outlooks become more likely to penalize target countries’ negative actions by reciprocating in kind (in both experiments) and, less sensitive toward the cooperative signals of other countries (UN experiment).

### Reciprocity and Individual Preferences on Foreign Policy

A long-held tradition on ethics stipulates that reciprocity is one of the prerequisites of moral behavior (Neusner & Chilton, 2008). Social psychologists concur that humans tend to have a moral intuition to reciprocate in their social interactions and associate reciprocity with ‘fairness’ and ‘equity’ (Fehr & Gachter, 2000; Goulder, 1960). For instance, people have an inclination to smile back when one smiles at them or retaliate when they are hit (e.g., Scharlemann et al., 2001).

An extensive literature on economic behavior establishes that both negative reciprocity—in ultimatum games (e.g., Hoffman et al., 1996)—and positive reciprocity—in trust or gift exchange games (e.g., McCabe et al., 1996)—condition individuals’ economic decisions. These studies also find that in small, experimental settings, individuals display a greater inclination to penalize uncooperative behavior than reward positive behavior (Charness & Rabin, 2000; Fehr & Gachter, 2000).

Although reciprocity may be a potent factor in regulating interpersonal relations, when individuals evaluate foreign policy proposals, their inclination to reciprocate in response to other countries’ actions may lose its prominence. On the one extreme, public sensitivity to certain issue areas such as human rights or the environment may lead citizens to favor normatively driven, unconditional policy options regardless of the policy choices of other countries. On the other extreme, citizens may evaluate international relations from the neorealist lenses of Realpolitik and refrain from responding in kind to the favorable policy actions of other countries when they are concerned that their countries would end up relatively worse off and/or the other party is perceived as a threat to national security.

This public ambivalence toward reciprocity in foreign policy has been exposed empirically. In a conjoint experiment on Swiss citizens, Rudolph et al. (2019) show that to cope with the social and environmental impacts of transnational business activity, citizens prefer unilateral regulations of home-country multinational firms operating abroad, despite their competition crippling effects. Similarly, in another study, voters in the US were found to ignore other countries’ green policies while evaluating their home countries’ climate change legislations (Tingley & Tomz, 2014).

On the other hand, several studies also concur that individuals’ foreign policy preferences are closely influenced by other countries’ policy decisions, although not necessarily in cooperative directions. In bilateral conflicts, the public in democracies prefers leaders to respond to an opponent’s cooperative or hostile actions in the same manner, though they are wary of extreme responses in either direction (McGinnis & Williams, 2001). Chilton (2015) finds that voters are more likely to comply with international laws on war when they expect reciprocal behavior from the opposing party. Similarly, Flavin and Nickerson (2009) note that when informed on the American practice of torture, respondents become more tolerant of torture by other nations. With regard to economic affairs, Chilton et al. (2020) maintain that in both the US and China, respondents are more likely to disapprove of foreign acquisitions if the foreign firm’s home country does not grant the same type of access for foreign companies.

### Reciprocity and Individual Attitudes on IOs and Trade

International organizations and trade agreements are two manifestations of international cooperation, and public support—or non-support—for them derive largely from common sources. A line of research explains micro-level support for international institutions and trade liberalization by focusing on ideational dispositions such as cosmopolitanism (e.g., De Wilde et al., 2019), internationalism (e.g., Kaltenhaler & Miller, 2013), national pride and threat perceptions (e.g., McLaren, 2002), or economic nationalism (Clift et al., 2012). Accordingly, individuals who are culturally open, younger, and hold more cosmopolitan and less nationalist values are more willing to utilize cooperative mechanisms and support free trade and the delegation of decision-making capabilities to IOs.

The second line scrutinizes the expected egotropic (Bearce & Scott, 2019; Gabel & Whitten, 1997) and sociotropic benefits (e.g.,Dellmuth & Tallberg, 2015; Mayda & Rodrik, 2005) of cooperation. In a nutshell, egotropic explanations posit that those with higher factor endowments in developed countries are more likely to consent to IOs and trade liberalization because the lifting of barriers will increase demand for their skills, consequently making them better off. Sociotropic explanations concur that support for free trade and cooperation through IOs is highly contingent on individuals’ assessments of the potential political or economic consequences on their communities either at the local or national level.

Both of these explanations, either focusing on ideational dispositions or economic self-interest, study the predictors of individual support for cooperation as a constant and largely ignore the complex interactions between participating countries in shaping popular opinion. However, how states and their voters approach cooperation is also a function of how their counterparts act. An individual’s ideational orientation or material self-interest might positively drive their general endorsement of global governance; however, if partner countries defect or exhibit relative gain-seeking behavior, then support for specific cooperation could substantially evaporate. This study contributes to the literature, therefore, by exploring the extent to which voters revise their preferences on political and economic cooperation *in response* to other states’ actions and how individual-level assessments moderate their response.

At the macro level, contributions to international institutions and trade liberalization essentially entail collective action problems. In the absence of enforcement mechanisms, the self-interest of any actor is to defect, either with the expectation of free riding, or minimize the risk of being exploited by the other party. Reciprocity partially helps overcome this dilemma; when assured of future in-kind response from the other participants, the incentive to cooperate grows, while the inclination to defect declines. As such, the principle of reciprocity has constituted a core pillar of the foundation of international institutions and served as a key principle in bilateral and multilateral trade liberalization (e.g., Bagwell & Staiger, 2001; Ruggie, 1992).

Policymakers do not act in a vacuum but are bound to act within the limits of public preferences. Specifically, public opinion is found to have a direct impact on IOs’ legislative decisions (Hagemann et al., 2017), the legitimacy and effectiveness of their policies (Dellmuth & Tallberg, 2015; Edwards, 2009), and the type and amount of funding they receive from donors (Bayram & Graham, 2017). Similarly, electoral preferences and sentiments on various aspects of international trade substantially shape legislative voting on trade policies (Hiscox, 2002; Milner & Tingley, 2011), the scope and depth of negotiated trade agreements (Young, 2017), and the lifecycle of trade negotiations (Eliasson & Huet, 2018).^1^

The close association between government actions and constituent preferences indicate that policymakers’ willingness to reciprocate another country’s actions on matters of foreign policy should be in line with voters’ motivations. Thus, *ceteris paribus*, voters are expected to respond positively to the cooperative actions of other parties and push their governments to retaliate or penalize non-compliance and defection.

First, strategically, policymakers and voters alike would consider reciprocity as a self-enforcement mechanism of cooperative arrangements that makes the provision of public goods of UN regime or mutual trade gains more likely. Moreover, international cooperation entails multiple, observing players. The decision of non-cooperation in response to cooperative signals by others can incur reputational costs for the home country and risk future retaliation by other countries. The pressure to cooperate would be particularly pronounced if the home country has an active presence in various IOs (Mitchell & Hensel, 2007).

Two, morally, reciprocal behavior yields positive judgments about the fairness of a country’s interactions with others (Brutger & Rathbun, 2021; Powers et al., 2022). Research has established that humans have a psychological preference for fairness and prefer decisions that minimize inequity in payoffs (Fehr & Schmidt, 1999). Concerns over relative contributions and shares are particularly pronounced in the distributional settings of IO membership and trade agreements, where asymmetrical provisions can not only shift the power balance among participant countries, but, equally important, raise concerns over its equity and equality of the outcome (e.g., Gowa & Mansfield, 2004; Yeung & Quek, 2022). Hence, Brutger and Rathbun show, when evaluating trade relations, Americans’ attitudes are often driven by a sense of fairness, albeit an egoistic one, according to which they value equal distribution of concessions and benefits, but oppose agreements that leave their home country relatively behind (2021). Therefore, individuals would be expected to favor balanced agreements whereby their home countries and other parties contribute equally to IOs or deliver equally favorable terms to each other. Following this line of thought, when partner countries signal changes in their positions—either positively or negatively—individuals would be inclined to follow suit and reciprocate over concerns of fairness.

#### H1a

Cooperative (uncooperative) signals by other countries increase (decrease) voters’ support for cooperation through IOs.

#### H1b

Cooperative (uncooperative) signals by other countries increase (decrease) voters’ support for cooperation on trade.

Next, we propose, individuals’ attitudes on further cooperation and their inclination to reciprocate are closely conditioned by evaluations of their forthcoming material prospects.

Individuals who hold negative assessments of their economic futures tend to develop protectionist attitudes to protect themselves and their close circles (Jacobs & Matthews, 2012). When economic predictions are bleak, voters become more sensitive to budgetary expenses, how their tax money is being spent, and whether domestic government programs are sufficiently provided for (Abbott & Jones, 2020; Peacock & Wiseman, 1961). The benefits accrued by participating countries and their public by cooperating in international endeavors are often indirect and long term. Moreover, most citizens are poorly informed when it comes to foreign affairs and tend to conflate various cooperative frameworks at the international scale (i.e., IOs or cross-border flows) when thinking about foreign policy (Bearce & Scott, 2019). Specifically, they are often naïve about the material costs of international institutions and cooperation and grossly overestimate how much their countries contribute to such endeavors (Dellmuth & Tallberg, 2015; Guisinger,^ 2009^; Paxton & Knack, 2008). Given that lack of awareness, when evaluating international cooperation, they tend to take cues from their own personal economic situations (Bearce & Scott, 2019; Dellmuth et al., 2022).

Consequently, for actors with shorter time horizons, spending much-needed resources on IOs or securing trade deals with adverse effects on domestic job markets are viewed as less favorable options than allocating resources for pressing domestic purposes, such as bolstering welfare services or reviving economic growth that would provide direct, short-term benefits. For instance, the literature on foreign aid shows that during economic downturns at home, donors tend to cut back on aid (Dang et al., 2013). Such cuts are often demanded (and even championed) by voters: public support for development cooperation typically plunges when donor respondents experience personal financial difficulties (Heinrich et al., 2016). Similarly, during COVID-19, citizens who were worried about the pandemic’s economic consequences were much more likely to support aid cuts (Kobayashi et al., 2021).

Pessimistic material expectations may dampen individuals’ willingness to pay for international cooperation; however, how does this disinclination affect their tendency to reciprocate? Reciprocity links today’s actions to expectations of future benefits. When the game is played frequently, actors have a greater incentive to cooperate because they value the future payoffs from interactions more than the short-term benefits. Bleak expectations about the material conditions change this equation by shortening the “shadow of the future” (Axelrod & Keohane, 1985). Under uncertainty, when actors expect their relative conditions to deteriorate, they become more inclined to prefer short-term benefits that could be attained today by defecting at the expense of long-term—but potentially higher—gains that could be acquired from cooperation. Similarly, when actors focus on maximizing their short-term payoffs in preparation for tomorrow’s unfavorable circumstances, the cost they associate with defection will be devalued. Consequently, the more negative voters’ perception of their material well-being are, the less inclined they will be to display reciprocal behavior.

Negative material expectations may also trigger psychological motivations that inhibit reciprocal behavior. Previous research identifies equity as a potent psychological motivation that drives foreign policy positions among particular segments of populations, and these equity-oriented individuals tend to be more attentive to the costs of cooperation (Powers et al., 2022). Following this line, we expect economic worries to amplify concerns over countries’ relative shares of contributions. The equity principle would dictate that allocations for common goods should be in proportion to an actor’s capabilities. If actors anticipate their material conditions to deteriorate, hence shrinking their capabilities, they would likely find arrangements that ask parties to reciprocate each other’s cooperative actions as being unfair.

However, reciprocity is not necessarily symmetrical. Positive reciprocity denotes cases whereby an actor’s favorable action is followed by a favorable response by the other actor, whereas negative reciprocity signifies negative interdependence, where a hostile action is met by a hostile reaction by the other party. When conditioned by positive reciprocity, actors become more likely to cooperate if they observe the other members contributing. When conditioned by negative reciprocity, actors avoid defection from cooperation because they anticipate their uncooperative behaviors to be penalized by in-kind responses from the other party.

Empirically, individual evaluations on future material conditions should yield contrasting micro-level attitudes in response to other countries’ positive and negative foreign policy signals. When other countries signal cooperative actions, voters with negative future assessments will considerably deviate from those with positive assessments. Given that they prefer immediate, low-return gains over long-term, high-return gains, we would expect those with pessimistic material expectations to be more likely to defect from cooperation and not reciprocate cooperative behavior. Conversely, when other countries signal uncooperative behavior, voters with negative material assessments will be more likely to counter defection with non-cooperation compared to those with positive expectations. From this argumentation, we extract two testable hypotheses on the moderating role of future expectations on reciprocal behavior:

#### H2a

Individuals with negative assessments of their material prospects are less likely to reciprocate cooperative behavior by other countries.

#### H2b

Individuals with negative assessments of their material prospects are more likely to reciprocate uncooperative behavior by other countries.

## Experimental Design: Case Selection

Our arguments may be limited by geographical scope conditions. Citizens might have varying rationales and willingness in displaying reciprocal behavior in different country contexts. For instance, in weaker countries, defection and free-riding could be more viable options to offset power imbalances, particularly when dealing with stronger opponents. Similarly, the social cultural norms and sense of fairness embedded in different polities might affect the way individuals approach the norm reciprocity. In settings where interpersonal and institutional trust is weak, social incentives to uphold reciprocal behavior are generally less effective (e.g., Carlin & Love, 2013). Despite these theoretical possibilities, with a few exceptions (e.g., Stroik et al., 2019), we lack evidence on the extent to which the norm of reciprocity conditions political behavior in cross-cultural contexts.

This study draws data from structurally two dissimilar countries. The US and Turkey differ substantially in terms of political regime, culture, development level as well as the level of institutional trust toward IOs. Despite their differences, politicians in both countries often cue voters about other countries’ policy actions when mobilizing support for their policy preferences on international obligations. Former US President Trump repetitively expressed his frustration with the unequal burden the US has to bear in financing IOs. When responding to the UN budget crisis in 2019, he announced “make all member countries pay, not just the United States.^2^” This discourse on reciprocity was even more explicit on US’ trade relations. In 2018, Trump declared that “from now on, we expect trading relationships to be fair and to be reciprocal.^3^” Similarly, Turkish President Erdogan has regularly urged for a revision of the Customs Union agreement with the EU on the grounds of fairness and reciprocity^4^ and blamed countries that fail to pay their membership dues in the Organization of Islamic Cooperation (OIC) for their lack of unity.^5^

The small-N of macro-level units in our research design does not allow us to test competing explanations on country-level differences in voters’ attitudes toward reciprocity. For such purposes, large-N cross-country designs with nationally representative samples would be needed. At the same time, attaining similarly robust treatment effects in two highly dissimilar country cases would indicate greater external validity and broader generalizability of our theoretical arguments.

## Methodology

The data for the empirical analysis of this paper is obtained through an online survey experiment fielded in October–December 2020 on a sample of 1533 adult respondents in the US and 1512 in Turkey.

The respondents in the US survey are recruited from the Amazon MTurk and, for the Turkish survey, from the panel of international online research company, Twentify. Given the circumstances of the pandemic, online surveys present practical advantages and minimize nonresponse bias. On the negative side, however, online panels may not be perfectly representative of certain groups. To improve representability, for both samples, quotas were applied based on age, gender and education to match the statistics of the general population.^6^ Several measures were taken to increase data quality. First, in both surveys, some attention-check questions were posed in the survey, and data on those who failed them were excluded. Second, in the American survey, only MTurkers with 5000 completed HITs and over 97% approval rates were employed. Finally, third, the geolocations of IP addresses were controlled to eliminate bots and non-US responses, who mask behind virtual private servers . Of the final sample in the US, the mean age was 43 (population mean μ = 38.1), 36.4% had college degrees (μ = 37.9), and 50% were women. Politically, 49.8% of the American respondents were aligned with the Democratic Party, 32.5% with the Republican Party, and 18.7% considered themselves independents.

For the Turkish survey, our sample was randomly drawn from the Twentify panel within the required target quotas. To prevent fraudulent responses, we relied on advanced features offered by the company, including profiling data to detect patterns, secure APIs, and unique survey URLs. Of the Turkish sample, the mean age was 34.3 (μ = 31.5), 30.1% had college degrees (μ = 21), and 50% were women.

To test our hypotheses, we conducted two vignette experiments in randomized order. The UN experiment involved a short description of a group of countries declaring their positions on funding the UN in the coming year. In the trade experiment, the hypothetical countries are said to announce their positions on tariff rates vis-à-vis survey country exports. In both designs, we varied two factors. The first and primary dimension of interest manipulated the content of the signal (*policy treatment*). The factor levels ranged from reducing contributions or tariff levels by a relatively large, 50%, relatively minor, 10%, keeping them as they are, increasing them by 10%, or 50%.

The second dimension of the vignette considered the signaling country characteristics (*signal source*). Methodologically, adding partner characteristics enhances the realism of the scenarios by presenting respondents a fuller picture about the policy vignette they are asked to evaluate. Moreover, manipulating signal source allows us to investigate whether the treatment of policy signals significantly vary across different partner country profiles. Previous research confirms that voters pay attention to how their countries fare vis-à-vis partner country when they assess the expected benefits and costs of cooperation (e.g., Mutz & Kim, 2017). Arguably, who the partner country and how positive its image to the in-group is, and how competitive the bilateral relations are might change these assessments. For instance, having experimentally manipulated the identity of the trading partner and relative gains, Herrmann et al. (2001), found that American citizens are most favorable of protectionism when the trading partner is a wealthy enemy and gains relatively more. Conversely, reciprocity is more likely among similar pairs with lower conflict of interest. (Axelrod & Keohane, 1985). Thus tentatively, we would expect voters to be more cooperative and motivated by positive reciprocity toward countries they evaluate positively. Accordingly, we varied the signaling countries based on their economic development levels, military power, religious identity and bilateral relations with the survey country. This dimension also included a baseline factor, with no information provided on the signaling countries.

Table 1 summarizes the two experimental treatment and their levels.

**Table 1:** Treatment levels

Policy Treatment (UN experiment)	reduce their contributions by 50% / reduce their contributions by 10%/ maintain their existing contributions/increase their contributions by 10%/increase their contributions by 50%
Policy Treatment (Trade experiment)	reduce the tariff rate against [survey country] exports by 50%/ reduce the tariff rate against [survey country] exports by 10%/ maintain the existing tariff rate on [survey country] exports / increase the tariff rate against [survey country] exports by 10%/ increase the tariff rate against [survey country] exports by 50%
Signal Source Treatment	[No information]/ ally/competitor of [survey country]/developed/underdeveloped/ militarily strong/militarily weak/ Christian (for the US) or Muslim (for Turkey)

Because the full factorial combination of two dimensions with 8 and 5 levels results in a vignette population of 40 different vignettes and renders a conventional vignette survey impractical, we adopt a factorial design by randomly selecting subpopulations of vignette sets for each respondent. Unlike single experiments, factorial vignette experiments do not suffer from low external validity due to their higher representativeness of substantive issues (Auspurg and Hinz 2014). A fully randomized selection of vignettes often yields orthogonal designs that minimizes confounding effects and enables to estimate the main and interaction effects (Steiner & Atzmüller, 2016). Accordingly, in our experiments, the vignette treatments were fully randomly assigned, so that the values of attributes were approximately uniformly distributed across resulting vignette tasks. Each respondent was asked to evaluate four unique combinations (tasks) for each vignette, resulting in around 6000 observations for each experiment per country.

The vignette on UN contributions was worded as follows:

A group of ______ (*one of the 8 country treatments in Table **1** randomly assigned*) countries has announced it will ______ (*one of the 5 policy treatments in Table **1** randomly assigned*) to the UN.

The US/Turkey should

- Reduce its contributions to the UN
- Maintain its existing contributions
- Increase its contributions to the UN

If respondents chose the options to reduce or increase, a following question asked them how much less (or more):

- Less than 10%
- 10%
- Between 11 and 49%
- 50%
- More than 50%

After reminding the respondents that the two vignettes are independent of each other, the trade vignette posed the following scenario:

A group of ______ *(one of the 8 country treatments randomly assigned)* countries has announced it will ______ *(one of the 5 policy treatments randomly assigned):*

The US/Turkey should:

- Reduce its tariff rates against these countries’ exports
- Maintain its existing tariff rates
- Increase its tariff rates against these countries’ exports

As in the UN experiment, a consecutive question with the exactly the same answer categories popped up for those who choose either the “increase” or “reduce” option to specify the degree. Figure A1 in the Online Appendix illustrates an example question for each vignette.

### Study Variables

For each experiment, the combined answers for the two subsequent questions constitute the first dependent variable, *cooperation*, to test H1a and H1b. *Cooperation* entails 11 answer categories, ranging from 1 = decrease (contributions) more than 50%, through 6 = maintain existing levels, to 11 = increase (contributions) more than 50% for the UN experiment. For the trade experiment, it ranges from 1 = increase (tariffs) more than 50% to 11 = decrease (tariffs) more than 50%. The five-level experimental treatment on foreign policy actions of the signaling countries (*Policy Treatment)* constitutes the independent variable of H1a and H1b.

To test whether negative future assessments moderate individuals’ tendency to reciprocate (H2a&H2b), we create a new binary variable, *reciprocity,* that covers observations in which respondents mirror the signal of other countries, though not necessarily match in degree (Kollock, 1993). Note that instead of strict tit-for-tat, we adopt “relaxed reciprocity” as our primary variable of interest, because policy negotiations on international cooperation through institutions or trade often do not involve explicit calculations of magnitude, as this would risk bargaining deadlocks (Axelrod & Keohane, 1985). For instance, Finger et al. (1999) document that during GATT’s Uruguay Round negotiations, negotiators never aimed to acquire perfectly matched benefits for their concessions, but instead accepted substantial variance between offers and returns.

For operationalization, respondents who react positively (i.e., increase contributions/tariffs by any degree) to positive treatments (i.e., signaling countries increase contributions/tariffs by 10 or 50%); react negatively (i.e., reduce contributions/tariffs by any degree) to negative treatments (i.e., signaling countries reduce contributions/tariffs by 10 or 50%); or remain neutral to neutral treatments (maintaining contributions/tariffs as they are) are considered to have reciprocated and are thus coded 1, and the rest, 0.

The independent variable to test H2a and H2b is an interaction term between the treatment variable, *Policy Treatment,* and respondents’ future material expectations. For the second component of this interaction, we rely on the following item: “How do you expect your economic conditions to change in the next 6 months?” (from 1 = much better to 5 = much worse).

The multivariate models include several covariates to increase the precision of the estimates for the treatment effects. Standard demographic controls are age, gender and education. Higher educated and younger individuals generally hold more cosmopolitan views and are more supportive of international cooperation (e.g., Inglehart, 1997). Two attitudinal variables, confidence in the UN and support for international trade account for respondents’ general position on political and economic integration.^7^ Table-A-2 reports the question wordings and summary statistics for all the covariates.

### Analysis

We first present two-way tables of frequency counts for four variants of our experiments (US-UN, US-Trade, Turkey-UN, Turkey-Trade) in Online Appendix B-1. The frequency distribution leads to some notable observations: First, in all four variants, regardless of the treatment, the most popular option is *keep as is*, suggestive of a status-quo preference among respondents. Subsequently, again in all four, the second-most frequently chosen option matches both the direction and the degree of the signal, e.g., *decrease 50%* when provided with *decrease 50%* signal. In conjunction, third, the policy treatment seems to substantially affect cooperative attitudes, as we proposed. In the UN experiment, 38.8% of the observations from the American sample and 34.4% from the Turkish one exhibit reciprocal behavior, namely becomes more cooperative in response to positive signals and less cooperative in response to negative signals. In the trade experiment, the average inclination to reciprocate is even higher: in 47.7% of American and 57.6% of Turkish observations, respondents’ cooperation preferences emulate the direction of the policy treatment.

Moving from descriptive findings, we next analyze if the policy signals exert statistically significant effects on individual responses. Because vignette tasks are nested within individuals, we run multilevel regression models with random intercepts and robust SEs at the individual level. Multilevel models are particularly suited for factorial vignette designs, as they allow us to estimate separate individual intercepts and—if desired—slope values that reflect divergences in individual attitudes (). More importantly, multilevel models enable us to gauge the effects of the vignette dimensions and respondent characteristics, including demographic and attitudinal ones, simultaneously within the same regression model, as we aim for in this study.

**Fig. 1:**
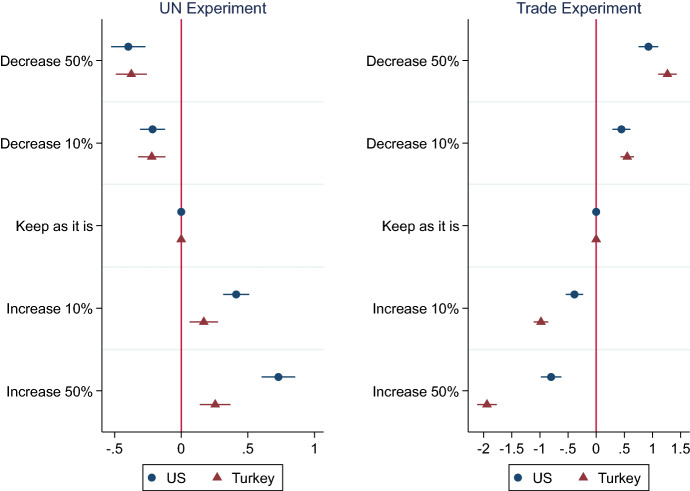
Marginal effects of the policy treatment (5-cat) on support for cooperation. The x-axis presents the coefficient estimates for study variables on the y-axis. Horizontal lines indicate 95% robust confidence intervals; points without lines indicate the reference categories. Experimental treatment on country effects are included in the model, but not reported in the figure

Figure 1 plots the marginal effects of mixed-effects linear regression that measures the main treatment effects on support for *cooperation*. The dots denote the median estimates, while the horizontal lines specify the 95% confidence intervals. For each categorical variable, the effect sizes are compared to the baseline, reference level, namely “*keep as is*.”

As hypothesized, in both countries, respondents are highly sensitive to the actions of other countries when deciding for cooperation through the UN and on trade. If the other countries reduce their contributions or their tariff rates vis-à-vis the survey country, the sample respondents respond in kind and vice versa. In short, reciprocity plays a fundamental role in respondents’ foreign policy attitudes in both these issue areas.

Although the treatment effects are strong across issue areas in both samples, the effect sizes considerably diverge between the US and Turkey. In the US, survey respondents reward the cooperative behavior of other countries significantly more than they punish uncooperative behavior. For instance, in the UN experiment, changing the treatment from the base level, *keep as is,* to *decrease 50%* reduces the average response on 11-point *cooperation* outcome by 0.43 points [− 0.57, − 0.30], yet its symmetrical opposite, *increase 50%,* rises the mean by 0.75 points [0.61, 0.88]. Moreover, in this experiment, compared to Turkish respondents, Americans become significantly more cooperative in response to the positive (increase contributions) signals of other countries.

In contrast, respondents in Turkey penalize the uncooperative behaviors of other countries more harshly than they reward cooperative behavior, particularly so in the trade experiment. When provided with the uncooperative *increase tariffs 50%* signal, the Turkish respondents favor higher tariffs against target countries by 1.94 points [1.77, 2.11]; however, when provided with the *decrease 50%* treatment, they elect to reduce tariffs only by 1.26 point [1.43, 1.10]. Additionally, in the trade experiment, compared to their American counterparts, Turkish respondents are significantly more unforgiving toward uncooperative (increase tariffs) signals though, at the same time, they respond more positively to the “decrease 50%” signal compared to the US sample.

To check the robustness of the treatment effects and investigate the effects of individual-level characteristics more closely, in Table 2 we run a full model controlling for several demographic and attitudinal variables, including future economic worries.

**Table 2:** Support for international cooperation-multilevel estimates

DV: Cooperation	United States		Turkey	
	UN Vignette	Trade Vignette	UN Vignette	Trade Vignette
Variables	Model-1	Model-2	Model-3	Model-4
Decrease 50%	− 0.435***	0.960***	− 0.370***	1.266***
	(0.067)	(0.091)	(0.059)	(0.084)
Decrease 10%	− 0.194***	0.467***	− 0.219***	0.553***
	(0.048)	(0.082)	(0.052)	(0.062)
Increase 10%	0.401***	− 0.379***	0.170**	− 0.980***
	(0.051)	(0.079)	(0.055)	(0.066)
Increase 50%	0.708***	− 0.838***	0.258***	− 1.940***
	(0.066)	(0.095)	(0.059)	(0.089)
Economic worries	− 0.208***	− 0.058	− 0.136**	− 0.009
	(0.049)	(0.040)	(0.049)	(0.035)
Trust UN	0.573***		0.460***	
	(0.039)		(0.048)	
Free Trade		0.198***		
		(0.038)		
Trust MNCs				0.023
				(0.034)
Same religion	0.035	0.022	0.130	0.148
	(0.072)	(0.118)	(0.077)	(0.088)
Ally	− 0.045	0.216	− 0.145*	0.283**
	(0.077)	(0.121)	(0.068)	(0.092)
Rival	0.146	− 0.001	− 0.035	− 0.273**
	(0.075)	(0.118)	(0.069)	(0.090)
Developed	− 0.017	0.072	− 0.146*	0.296***
	(0.076)	(0.117)	(0.073)	(0.087)
Underdeveloped	0.171*	0.318**	0.081	− 0.159
	(0.073)	(0.114)	(0.078)	(0.099)
Military Strong	− 0.002	0.066	− 0.106	− 0.030
	(0.075)	(0.122)	(0.070)	(0.085)
Military Weak	0.087	− 0.042	0.083	0.279**
	(0.071)	(0.116)	(0.070)	(0.088)
Demographic controls	Yes	Yes	Yes	Yes
Constant	4.754***	5.385***	6.023***	6.143***
	(0.305)	(0.280)	(0.385)	(0.274)
Individual-vignette intercept	0.30***	0.25**	0.55***	0.07
	(0.03)	(0.09)	(0.02)	(0.05)
Observations	5824	5824	6016	6041
Number of groups	1456	1456	1512	1512

Robust standard errors in parentheses. ***p < 0.001, **p < 0.01, *p < 0.05. Baseline categories are “keep as is” for Policy treatment and “no information” for signal source treatment. Same religion is Christian for the US, Muslim for Turkey

Expectedly, the effects of policy treatment are robust in the presence of control variables. Any changes from the base level significantly shifts support for cooperation in the direction of change. The country treatment also attains some statistical significance. In the American sample, in both experiments, when respondents are provided with a signal originated from poorer countries, they become more supportive of cooperation compared to the base category of no information provided “group of countries.” Turkish respondents, on the other hand, are favorable of tariff liberalization with allies, developed, and militarily weak countries, while they favor higher tariffs vis-à-vis rivals. Finally, we observe that in the UN experiment, those American and Turkish respondents who expect their economic welfares to deteriorate in the next six months are significantly less supportive of higher contributions to the UN, though their economic expectations do not wield any influence on their support for trade liberalization.

**Fig. 2:**
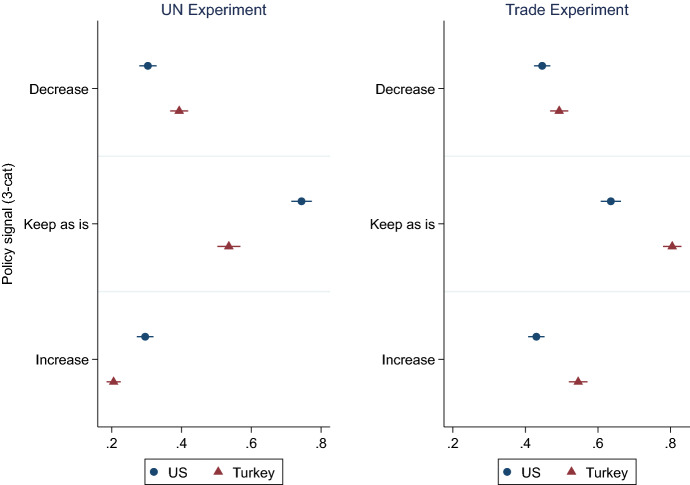
Predictive margins of policy signal (3 categories) on reciprocity. The x-axis presents the predicted margins of categorical Policy signal treatment (3-cat.) on the dependent variable, reciprocity, for the US and Turkey, based on multilevel estimates. Horizontal lines indicate 95% robust confidence intervals

**Fig. 3:**
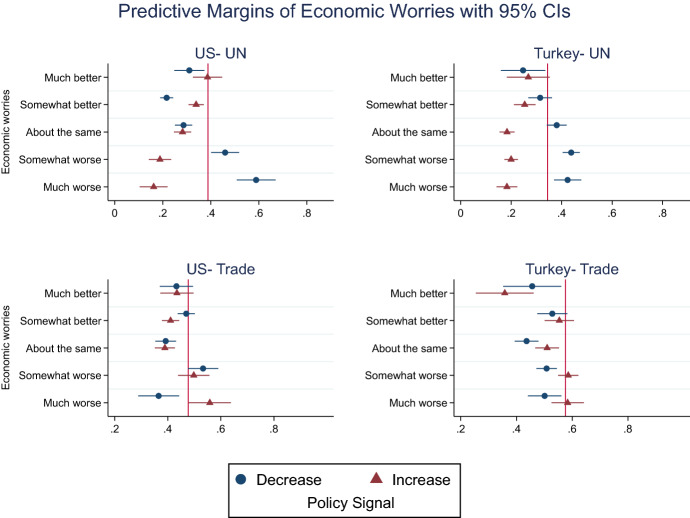
Predictive margins of economic worries conditional on policy signal. The x-axis presents the predicted margins of Economic worries on the dependent variable, reciprocity, for Increase and Decrease categories of Policy signal treatment, based on multilevel estimates. Horizontal lines indicate 95% robust confidence intervals. The red line denotes sample mean

For a straightforward demonstration of policy signals on the inclination to reciprocate, we collapse the five-category *Policy Treatment* into three categories (decrease, keep as is, increase) and plot estimate the adjusted predictions on *reciprocity* for each treatment categories in the two sample countries (Figs. 2, 3). In other words, the estimates report the probability of reciprocating—namely, choosing the “decrease” option when treated by “decrease” signals, choosing the “increase” option when treated by “increase,” and “keep as is” option when treated by “keep as is” signal.^8^

The results exhibit several patterns: One, the strongest effects on reciprocal behavior is always caused by “keep as is.” When the other countries signal no changes in their positions, voters strongly prefer to maintain the status quo in both experiments and both countries. Second, particularly when cued by “decrease” and “increase” treatments, the inclination to reciprocate is considerably higher in the trade experiment than in the UN experiment. Third, in parallel to our findings on cooperation, in the UN experiment, the American respondents are significantly less likely to reciprocate toward negative (decrease) signals and are more likely to reciprocate toward positive (increase) signals compared to the Turkish respondents. Fourth, in contrast, in the trade experiment, for all three types of signals, compared to the American sample, the Turkish respondents are significantly more likely to emulate the partner signal.

We have shown that respondents are generally sensitive to the actions of partner countries and tend to reciprocate on matters of political and economic cooperation. However, do future assessments affect individuals’ tendency to reciprocate, as hypothesized in H2a and H2b?

To test this, in Figure 3 we plot the conditional effects of the two policy treatments, “increase” and “decrease,” on the outcome variable, *reciprocity,* across the range of economic worries (the corresponding regression results as well as mean-comparison tests are in Online Appendix A-3 and A-4). Substantially, the plot estimates report the predicted probability of reciprocating for respondents with different economic expectations when manipulated by positive or negative signals. In each subgraph, the red line denotes the sample mean of the outcome variable.

The results show that in line with our hypotheses, positive and negative signals exert significantly diverging effects for respondents with economic worries. In both experiments, those who expect their economic conditions to improve either do not react differently to the “increase” and “decrease” signals or are more likely to reward positive signals than they are to punish negative signals (as in the case of Americans who expect “somewhat better” conditions). Yet, we observe the opposite tendency among those with pessimistic economic assessments.

In the UN experiment, when other countries signal an increase in their contributions, only between 15 and 20% of these pessimist individuals in either country reciprocate positively and consent to higher contributions. However, when other countries cut their contributions, over 43% choose to follow suit and penalize defection. Moreover, the pessimistic individuals’ inclination to reciprocate starkly contrast with those of optimists, as we proposed. For instance, compared to Americans who expect “much better” economic conditions, respondents with “much worse” expectations are 23.7% less likely to reciprocate with increased contributions, but 30.6% more likely to match “decrease” signals, both significant at 0.001 level. We observe a similar trend among the Turkish respondents: Compared to the most optimist groups, the most pessimists are 8.6% (*p*-value 0.051) less likely to respond to positive (increase contributions) signals, but 17.6% (*p*-value 0.002) more likely to respond in kind toward negative (decrease) signals. Substantially, the results of the UN experiment lend ample evidence for both H2a and H2b.

For the trade scenario, a largely symmetrical picture emerges. With the exception of “somewhat worse” in the US, for those with economic worries in both countries, the predicted probability of reciprocating the positive signals of reduced tariff barriers is significantly lower than the mean. Along the same line, when informed about noncooperation—that is rising tariffs against home country-, the same groups’ predicted probability of reciprocating rises to over 50%. Equally informative, we observe significantly different treatment effects between those on the opposite ends of economic expectations. When cued with a positive “decrease” (trade barriers) treatment, these two groups do not differ in their inclination for reciprocity, unlike what we proposed in H2a. Conversely, confirming what we expected in H2b, when cued by a negative “increase” (tariffs) treatment, those who expect “much worse” conditions are significantly more likely to reciprocate than those who expect “much better” conditions—by 12.5% in the US (*p*-value = 0.11) and 21.8% in Turkey (*p*-value = 0.000).

In the Online Appendix A-5, we also chart the average marginal effects of economic worries plotted for each category of target action. For the UN vignette, in both samples, the marginal effect of economic worries is positive and statistically significant in reducing contributions by the target countries, as the 95% confidence intervals do not cross zero. In other words, when other countries reduce their UN dues, each unit increase in economic pessimism significantly increases the probability of the respondents reciprocating and opting to lower contributions. On the other hand, when other countries increase their dues—or keep them as they are in the US experiment—economic pessimism reduces the likelihood of reciprocal behavior, and respondents become critical of contributing more. In the trade experiment, the marginal effects of economic worries are statistically significant for the target actions of “increasing” tariff barriers; in the event of rising trade discrimination against national exports, economic pessimism makes respondents more inclined to retaliate.

These results establish that individuals’ negative material assessments do not necessarily cause them to reciprocate less on average. In fact, when other countries signal uncooperative behavior, those with negative future assessments become more likely to reciprocate by displaying uncooperative behavior themselves, a finding robustly present in both issue areas and both samples. However, pessimistic expectations at the micro level make individuals less sensitive to the positive signals of other countries and hence less likely to reciprocate as we proposed and found evidence in our UN experiment. In other words, negative prospective expectations induce individuals to become more unforgiving of negative actions and less generous toward the favorable actions of other countries.

### Signal Source Effects and Robustness Checks

The signal source treatment of our experimental design allows us to investigate the exact partner attributes that sway voters’ inclination to reciprocate. We present the findings of this complementary analysis in the Online Appendix (A-6). The results show that respondents in both countries display significantly positive attitudes toward militarily weak and underdeveloped countries such that they either are less likely to penalize their uncooperative behavior (US—both experiments) or more likely to reward their cooperative behavior (Turkey—trade experiment). Turkish respondents are also more likely to reciprocate the actions of Muslim countries that reduce their tariff barriers. The varying effects of country types on reciprocal foreign policy preferences may be driven by concerns over equity (i.e., varying contributions are acceptable as long as they are proportional to country capabilities) or cultural affinity (in the Turkish case). Caution is warranted against an over-interpretation of these country effects, however, as our design does not allow us to identify the causal mechanisms on why respondents are more sensitive to the signals of particular types of countries over others. Future research could provide greater illumination via investigation of signal source effects in driving inclination to reciprocate, with more fine-grained experimental designs customized for this purpose.

Two robustness checks are performed. First, to test H2a and H2b, we gauge individuals’ future assessments with an alternative question, in which respondents are asked how optimistic they are about their futures in general (from 1 = extremely optimistic, to 5 = extremely pessimistic), and rerun the models using general pessimism data and its interaction with the policy signal (A-7). Second, we repeat our analyses on the full sample including respondents that failed the attention check (A-8).^9^ Though some of the significance levels weaken, the results of both checks are fully in line with previous findings.

Additionally, we construct a variable called *strict reciprocity*, which denotes responses that only match both the direction and degree of the policy signal. 27.2% of Americans and 18% of Turks in the UN experiment, and 32.8% of Americans and 36.6% of Turks in the trade experiment followed this strictly rigid tit-for-tat strategy. Next, we scrutinize if prospective economic assessments cause respondents to respond to other countries’ signals by retaliating with the exact same magnitude as communicated in the signal. In two variants of our experiments—the UN experiment in the US and the trade experiment in Turkey—the results are extremely similar to the results obtained for relaxed reciprocity. In the UN experiment, Americans with negative assessments are less likely to mimic cooperative signals, though more so toward uncooperative signals. In the trade experiment, Turkish respondents are more likely to penalize uncooperative signals by preferring the exact same retaliation. In the other two variants, the results run parallel to our main findings, though considerably weaker in degree and present only for specific subgroups.^10^

## Conclusion

In this paper, we investigated if reciprocity motivates individuals’ preferences on international cooperation through IOs and on trade, and the extent to which these preferences are moderated by negative material expectations. For the empirical analysis, we relied on data acquired from two factorial vignette experiments fielded with over 3000 respondents in the US and Turkey. By manipulating other countries’ foreign policy decisions with respect to their contributions to the UN and tariff rates vis-à-vis survey country exports, randomly assigning participants into various treatment groups, and holding all other variables constant, we were able to isolate the independent effects of the treatment conditions.

The results exhibit the strong presence of reciprocity in driving individual preferences with respect to international cooperation. In both experiments, a substantial number of sample respondents reacted positively to the cooperative signals of other countries and negatively to uncooperative ones. At the same time, there were some variations in how respondents reacted to positive and negative signals across country samples. In the US, the degree of rewarding cooperative behavior by other countries was greater than penalizing uncooperative signals. In Turkey in contrast, respondents were more unforgiving of defection than they were of rewarding greater cooperation.

When the moderating role of individuals’ material assessments on their inclination to reciprocate are scrutinized, we observe that bleak evaluations of economic future produced strong shifts away from positive reciprocity toward negative reciprocity. Specifically, economic pessimism makes individuals significantly more likely to penalize target countries’ negative actions by responding in kind while impeding them from responding positively to targets’ positive actions. These results were robust for both country samples.

Empirically, we found that on two issue domains, namely contributions to the UN and bilateral trade relations, individual foreign policy preferences are substantially impacted by how other countries act. One potential policy implication of this finding is that leaders can persuade their electorate on particular domains of international cooperation by cueing them about the actions of other countries. However, given that bleak expectations of the future make individuals less responsive to the positive signals of other countries and more so to negative ones, populist leaders who seek to dissuade voters from being part of international community seem to have higher chances of success than those championing for deeper political and economic integration.

Our results contribute to the flourishing literature that explores the role of behavioral norms and social preferences in driving individual foreign policy attitudes (e.g., Brutger & Rathbun, 2021; Kreps & Maxey, 2017; Powers et al., 2022; Yeung & Quek, 2022). Speaking to this body of research, our findings suggest that the effect of such norms, i.e., reciprocity, is not uniform across individuals, but rather vary based on their future assessments. At the same time, our study identifies new avenues for future research. Though we assumed that individuals’ assessments of their own material futures affect their foreign policy attitudes by cueing them about the state of the economy in general and the budgetary implications of international cooperation, future studies could better disentangle the role of egotropic versus sociotropic dynamics and shed more light on the economic and psychological motivations. Further studies can also explore the extent to which the length of time horizon and preference for immediate payoffs attenuate the inclination to reciprocate positive signals. On a broader level, another avenue for research could be investigations into other individual-level characteristics such as personality traits, cognitive biases, partisanship, or general foreign policy dispositions that may affect the inclination to reciprocate.

## Supplementary Information

Below is the link to the electronic supplementary material.

Supplementary file1 (DOCX 188 kb)

## Data Availability

The data and corresponding replication files will be available.

## Footnotes

1 However, it should also be noted that some studies question the electoral connection between trade policy and voting decisions (e.g., Guisinger, 2009) or demonstrate that the effects of individual trade preferences on their voting behavior is conditional on government policies (e.g., Margalit, 2011).

2 https://eu.usatoday.com/story/news/politics/2019/10/09/donald-trump-dismisses-united-nations-deficits-says-others-should-pay/3917554002/.

3 https://trumpwhitehouse.archives.gov/briefings-statements/president-donald-j-trump-confronting-chinas-unfair-trade-policies/.

4 https://www.swp-berlin.org/publications/products/arbeitspapiere/CATS_Working_Paper_Nr_5_Doruk_Arbay.pdf.

5 https://www.hurriyetdailynews.com/erdogan-urges-islamic-leaders-to-pay-their-dues-97889.

6 For the MTurk, this was implemented through the feature of Premium Qualifications.

7 Due to the data availability, we had to use two different items to account for support for economic openness. In the US, the respondents are asked if the governments should encourage international trade, whereas in Turkey, they are probed on whether they trust in MNCs.

8 Corresponding regression tables on country-level differences in treatment effects for both dependent variables are available in Online Appendix B2.

9 This robustness check was only for the American sample, as we do not have recorded data for those who fail the attention checks in the Turkish survey.

10 For instance, in the UN experiment, those with somewhat worse expectations in Turkey are significantly more likely to match uncooperative signals compared to somewhat better expectations, as we proposed (6.5%, p-value 0.001). Yet, we do not observe any statistically different reaction toward decrease signals between those with “much better” and “much worse” expectations at 0.05 level, even though the effects are in the hypothesized direction (4.9%, p-value 0.116).
