# Supplementary material for: When to Not Respond in Kind? Individuals’ Expectations of the Future and Their Support for Reciprocity in Foreign Policy
Source: Polit Behav. 2023 Jan 16:1–23. Online ahead of print. doi: 10.1007/s11109-023-09857-y (PMC9841930; doi:10.1007/s11109-023-09857-y)
Supplement: Supplementary file 1 — Supplementary file1 (DOCX 188 kb) [file 11109_2023_9857_MOESM1_ESM.docx]

**Appendix**

**Table of Contents:**

1. A-1- Example questions
2. Table A-2: Study Variables
3. Table A-3a: Policy Signal-Economic Worries Interactions- Multilevel Logit Estimates -UN Experiment
4. Table A-3b: Policy Signal-Economic Worries Interactions - Multilevel Logit Estimates-Trade Experiment
5. Table A-4: Test of Mean Differences for Interaction Effects between Economic Worries and Policy Treatment
6. Figure-A-5: Average Marginal Effects of Economic Worries on Reciprocity across Policy Treatment (3 cat.)
7. Table-A-6: Policy Signal (3-cat)- Signal source Interactions: Multilevel Logit Estimates
8. Table A-7: Policy Signal- General Pessimism Interactions: Multilevel Logit Estimates
9. A-8: Main Models with full sample (US only) –including those who fail attention checks
10. Table A-9a: Interaction Effects on Strict Reciprocity- Multilevel Logit Estimates
11. Table A-9b: Test of Mean Differences across values of Economic Worries and Policy Treatment –Strict reciprocity
12. Table B-1 Frequency Distributions between Policy Signal and Cooperation
13. B-2 Country-level Differences in Treatment Effects

**A-1 Example questions**

**UN vignette**

**Trade vignette**

**Table A-2: Study Variables**

| **Variable/min-max** | **M** | **SD** | **Variable Explanation** |
| --- | --- | --- | --- |
| **Cooperation on UN** | US: 5.73  Turkey: 5.42 | US: 2.12  Turkey: 2.24 | 1=Decrease more than 50% to 11= Increase more than 50% |
| **Cooperation on Trade** | US: 5.95  Turkey: 5.80 | US: 2.39  Turkey: 2.32 | 1=Increase more than 50% to 11= Decrease more than 50% |
| **Reciprocity on UN** | US: 0.39  Turkey: 0.34 | US: 0.49  Turkey: 0.48 | 0=no reciprocity 1=reciprocity |
| **Reciprocity on Trade** | US: 0.48  Turkey: 0.58 | US: 0.50  Turkey: 0.49 | 0=no reciprocity 1=reciprocity |
| Economic worries | US: 2.64  Turkey:3.40 | US: 1.06  Turkey: 1.06 | From 1=much better, to 5= much worse |
| Age (18-99) | US: 42.98  Turkey: 33.95 | US: 13.96  Turkey: 11.05 |  |
| Gender (1-2) | US: 1.50  Turkey: 1.48 | US: 0.50  Turkey: 0.50 | 1=female, 2=male |
| Education (1-7) | US: 4.22  Turkey: 4.47 | US: 1.06  Turkey: 1.28 | US: from 1=No formal education, to 6=Master’s/PhD  Turkey: from 1=No formal education, to 7=Master’s/PhD |
| General Pessimism | US: 2.33  Turkey:2.90 | US: 1.05  Turkey: 1.19 | From 1=extremely positive, to 5= extremely negative |
| Trust UN | US: 3.00  Turkey: 3.01 | US: 1.28  Turkey: 1.10 | From 1=none at all, to 5= a great deal |
| Support Free Trade (US)/ Trust MNCs (Turkey) | US: 3.33  Turkey:2.79 | US: 1.05  Turkey: 1.09 | From 1=strongly approve, to 5= strongly disapprove |

**Table A-3a: Policy Signal-Economic Worries Interactions- Multilevel Logit Estimates**

**UN Experiment**

| **DV: Reciprocity** | **United States** | | **Turkey** | |
| --- | --- | --- | --- | --- |
| VARIABLES | Model-1 | Model-2 | Model-3 | Model-4 |
|  |  |  |  |  |
| Decrease | -2.372*** | -2.447*** | -0.967*** | -0.965*** |
|  | (0.250) | (0.255) | (0.205) | (0.204) |
| Increase | -2.396*** | -2.411*** | -2.036*** | -2.031*** |
|  | (0.228) | (0.234) | (0.199) | (0.199) |
| Much better | -0.787** | -0.884** | -0.506 | -0.482 |
|  | (0.303) | (0.327) | (0.354) | (0.360) |
| Somewhat better | 0.848*** | 0.799** | -0.287 | -0.308 |
|  | (0.249) | (0.253) | (0.218) | (0.219) |
| Somewhat worse | -0.161 | -0.171 | -0.384* | -0.379* |
|  | (0.300) | (0.302) | (0.184) | (0.185) |
| Much worse | -1.346*** | -1.277** | -0.419 | -0.454 |
|  | (0.383) | (0.394) | (0.247) | (0.247) |
| DecreaseXMuch better | 0.927* | 1.051* | -0.163 | -0.162 |
|  | (0.422) | (0.442) | (0.525) | (0.526) |
| DecreaseXSomewhat better | -1.298*** | -1.210*** | -0.022 | -0.025 |
|  | (0.327) | (0.332) | (0.317) | (0.316) |
| DecreaseXSomewhat worse | 1.084* | 1.118** | 0.636* | 0.616* |
|  | (0.426) | (0.433) | (0.267) | (0.267) |
| DecreaseXMuch worse | 2.914*** | 3.057*** | 0.609 | 0.604 |
|  | (0.553) | (0.568) | (0.348) | (0.349) |
| IncreaseXMuch better | 1.361*** | 1.144** | 1.020* | 1.020* |
|  | (0.400) | (0.423) | (0.509) | (0.510) |
| IncreaseXSomewhat better | -0.526 | -0.485 | 0.722* | 0.711* |
|  | (0.304) | (0.310) | (0.313) | (0.313) |
| IncreaseXSomewhat worse | -0.468 | -0.435 | 0.495 | 0.479 |
|  | (0.385) | (0.391) | (0.257) | (0.257) |
| IncreaseXMuch worse | 0.502 | 0.560 | 0.417 | 0.417 |
|  | (0.482) | (0.499) | (0.352) | (0.353) |
| Same Religion | -0.286* | -0.335* | 0.034 | 0.041 |
|  | (0.134) | (0.137) | (0.113) | (0.113) |
| Ally | 0.474*** | 0.466*** | 0.166 | 0.172 |
|  | (0.131) | (0.136) | (0.108) | (0.107) |
| Rival | -0.221 | -0.230 | -0.027 | -0.025 |
|  | (0.135) | (0.139) | (0.114) | (0.114) |
| Developed | 0.086 | 0.063 | 0.120 | 0.128 |
|  | (0.131) | (0.137) | (0.109) | (0.109) |
| Developing | 0.001 | -0.025 | -0.031 | -0.029 |
|  | (0.132) | (0.137) | (0.122) | (0.122) |
| Military Strong | -0.079 | -0.105 | 0.020 | 0.021 |
|  | (0.135) | (0.139) | (0.114) | (0.113) |
| Military Weak | -0.280* | -0.294* | -0.090 | -0.084 |
|  | (0.133) | (0.137) | (0.126) | (0.126) |
| **Demographic Controls** | **NO** | **YES** | **NO** | **YES** |
| Trust UN | 0.036 | 0.038 | -0.060* | -0.057 |
|  | (0.037) | (0.038) | (0.030) | (0.030) |
| Constant | 1.001*** | 1.483*** | 0.415** | 0.194 |
|  | (0.195) | (0.333) | (0.158) | (0.279) |
| Individual-vignette intercept | 1.115*** | 1.079*** | 0.310*** | 0.295*** |
|  | (0.138) | (0.139) | (0.077) | (0.076) |
| Observations | 6,132 | 5,824 | 6,016 | 6,016 |
| Number of groups | 1,533 | 1,456 | 1,512 | 1,512 |

*** p<0.001, ** p<0.01, * p<0.05 Baseline categories are “about the same” for economic worries, “keep as is” for Policy treatment, and “no information” for signal source treatment.

**Table A-3b: Policy Signal-Economic Worries Interactions - Multilevel Logit Estimates**

**Trade Experiment**

| **DV: Reciprocity** | **United States** | | **Turkey** | |
| --- | --- | --- | --- | --- |
| VARIABLES | Model-1 | Model-2 | Model-3 | Model-4 |
|  |  |  |  |  |
| Decrease | -1.400*** | -1.421*** | -2.516*** | -2.517*** |
|  | (0.178) | (0.180) | (0.269) | (0.267) |
| Increase | -1.414*** | -1.452*** | -2.095*** | -2.096*** |
|  | (0.173) | (0.175) | (0.251) | (0.249) |
| Much better | -0.643** | -0.686** | -1.066* | -0.945 |
|  | (0.237) | (0.252) | (0.492) | (0.487) |
| Somewhat better | -0.334 | -0.398* | -0.515 | -0.505 |
|  | (0.173) | (0.176) | (0.338) | (0.336) |
| Somewhat worse | -0.303 | -0.433 | -0.012 | -0.098 |
|  | (0.224) | (0.232) | (0.279) | (0.277) |
| Much worse | -0.286 | -0.396 | -0.372 | -0.499 |
|  | (0.296) | (0.303) | (0.364) | (0.371) |
| DecreaseXMuch better | 0.832** | 0.871** | 1.182 | 1.171 |
|  | (0.313) | (0.335) | (0.664) | (0.667) |
| DecreaseXSomewhat better | 0.691** | 0.697** | 1.041* | 0.978* |
|  | (0.226) | (0.229) | (0.415) | (0.414) |
| DecreaseXSomewhat worse | 0.946** | 1.026*** | 0.422 | 0.428 |
|  | (0.302) | (0.311) | (0.337) | (0.335) |
| DecreaseXMuch worse | 0.160 | 0.327 | 0.741 | 0.744 |
|  | (0.398) | (0.407) | (0.440) | (0.445) |
| IncreaseXMuch better | 0.854** | 0.780* | 0.182 | 0.128 |
|  | (0.318) | (0.338) | (0.586) | (0.583) |
| IncreaseXSomewhat better | 0.433 | 0.471* | 0.765 | 0.742 |
|  | (0.224) | (0.226) | (0.396) | (0.395) |
| IncreaseXSomewhat worse | 0.802** | 0.845** | 0.447 | 0.451 |
|  | (0.292) | (0.300) | (0.320) | (0.318) |
| IncreaseXMuch worse | 1.059** | 1.121** | 0.794 | 0.798 |
|  | (0.386) | (0.391) | (0.425) | (0.428) |
| Same Religion | -0.146 | -0.108 | -0.167 | -0.162 |
|  | (0.119) | (0.121) | (0.127) | (0.127) |
| Ally | -0.082 | -0.056 | -0.188 | -0.192 |
|  | (0.117) | (0.119) | (0.126) | (0.125) |
| Rival | -0.117 | -0.089 | -0.170 | -0.175 |
|  | (0.117) | (0.120) | (0.128) | (0.127) |
| Developed | -0.018 | -0.009 | -0.851*** | -0.857*** |
|  | (0.119) | (0.121) | (0.128) | (0.128) |
| Developing | -0.081 | -0.067 | -0.926*** | -0.921*** |
|  | (0.117) | (0.119) | (0.148) | (0.147) |
| Military Strong | 0.043 | 0.047 | -0.292* | -0.281* |
|  | (0.116) | (0.118) | (0.131) | (0.130) |
| Military Weak | -0.045 | -0.036 | -0.528*** | -0.537*** |
|  | (0.120) | (0.122) | (0.140) | (0.140) |
| **Demographic Controls** | **NO** | **YES** | **NO** | **YES** |
| Free Trade/ Trust MNCs | 0.077* | 0.078* | -0.067 | -0.065 |
|  | (0.034) | (0.033) | (0.047) | (0.047) |
| Constant | 0.961*** | -0.035 | 2.541*** | 0.903* |
|  | (0.157) | (0.257) | (0.237) | (0.393) |
| Individual-vignette intercept | 0.575*** | 0.531*** | 2.205*** | 2.067*** |
|  | (0.090) | (0.090) | (0.199) | (0.191) |
| Observations | 6,132 | 5,824 | 6,041 | 6,041 |
| Number of groups | 1,533 | 1,456 | 1,512 | 1,512 |

*** p<0.001, ** p<0.01, * p<0.05 Baseline categories are “about the same” for economic worries, “keep as is” for Policy treatment, and “no information” for signal source treatment.**Table A-4: Test of Mean Differences for Interaction Effects between Economic Worries and Policy Treatment**

| ***United States-UN*** | | | |
| --- | --- | --- | --- |
| ***Treatment*** | ***Difference*** | ***standard error*** | ***p-value*** |
| Much worse X increase (N=178) vs Much better X increase (N=293) | -0.237 | 0.042 | **0.000** |
| Much worse X decrease (N=166) vs Much better X decrease (N=240) | 0.306 | 0.047 | **0.000** |
| Somewhat worse X increase (N=311) vs Somewhat better X increase (N=996) | -0.162 | 0.029 | **0.000** |
| Somewhat worse X decrease (N=326) vs Somewhat better X decrease (N=1030) | 0.251 | 0.028 | **0.000** |
| ***United States-Trade*** | | | |
| ***Treatment*** | ***Difference*** | ***standard error*** | ***p-value*** |
| Much worse X increase (N=168) vs Much better X increase (N=269) | 0.125 | 0.049 | **0.011** |
| Much worse X decrease (N=170) vs Much better X decrease (N=270) | -0.068 | 0.048 | 0.154 |
| Somewhat worse X increase (N=302) vs Somewhat better X increase (N=987) | 0.086 | 0.032 | **0.008** |
| Somewhat worse X decrease (N=331) vs Somewhat better X decrease (N=1028) | 0.066 | 0.032 | **0.037** |
|  |  |  |  |
| ***Turkey-UN*** | | | |
| ***Treatment*** | ***Difference*** | ***standard error*** | ***p-value*** |
| Much worse X increase (N=354) vs Much better X increase (N=110) | -0.086 | 0.044 | **0.051** |
| Much worse X decrease (N=347) vs Much better X decrease (N=98) | 0.176 | 0.055 | **0.002** |
| Somewhat worse X increase (N=908) vs Somewhat better X increase (N=420) | -0.052 | 0.024 | **0.033** |
| Somewhat worse X decrease (N=876) vs Somewhat better X decrease (N=401) | 0.127 | 0.029 | **0.000** |
|  |  |  |  |
| ***Turkey-Trade*** | | | |
| ***Treatment*** | ***Difference*** | ***standard error*** | ***p-value*** |
| Much worse X increase (N=342) vs Much better X increase (N=97) | 0.218 | 0.057 | **0.000** |
| Much worse X decrease (N=330) vs Much better X decrease (N=106) | 0.066 | 0.056 | 0.238 |
| Somewhat worse X increase (N=897) vs Somewhat better X increase (N=434) | 0.034 | 0.029 | 0.245 |
| Somewhat worse X decrease (N=900) vs Somewhat better X decrease (N=413) | -0.031 | 0.030 | 0.304 |

Note: The t statistic is obtained from a *t* test comparing selected treatment groups with symmetrically opposite economic expectations. *p*-values in bold denote statistical significance at 0.05 level.

**Figure-A-5: Average Marginal Effects of Economic Worries on Reciprocity across Policy Treatment (3 cat.)**

******

***Note:*** *The x-axis presents the average marginal effects of Economic worries on the dependent variable, reciprocity, for three categories of Policy signal treatment on the y-axis, based on multilevel estimates. Horizontal lines indicate 95% robust confidence intervals.*

**Table-A-6: Policy Signal (3-cat)-Signal Source Interactions: Multilevel Logit Estimates**

| **DV: RECIPROCITY** | **United States** | | **Turkey** | |
| --- | --- | --- | --- | --- |
| VARIABLES | **UN** | **Trade** | **UN** | **Trade** |
| Decrease | -2.090*** | -0.938*** | -0.422* | -2.657*** |
|  | (0.257) | (0.227) | (0.212) | (0.334) |
| Increase | -2.305*** | -0.718** | -1.631*** | -1.806*** |
|  | (0.264) | (0.227) | (0.228) | (0.333) |
| Same religion | -0.074 | 0.184 | 0.031 | -0.817* |
|  | (0.307) | (0.268) | (0.241) | (0.380) |
| Ally | 0.352 | -0.129 | 0.354 | -0.383 |
|  | (0.313) | (0.269) | (0.247) | (0.391) |
| Rival | -0.077 | -0.481 | 0.006 | -0.579 |
|  | (0.302) | (0.260) | (0.244) | (0.398) |
| Developed | 0.196 | 0.268 | 0.416 | -0.958* |
|  | (0.304) | (0.271) | (0.255) | (0.383) |
| Underdeveloped | -0.132 | 0.112 | 0.072 | -0.925* |
|  | (0.304) | (0.262) | (0.248) | (0.385) |
| Military Strong | -0.087 | 0.172 | 0.175 | -0.986* |
|  | (0.306) | (0.260) | (0.243) | (0.384) |
| Military Weak | 0.285 | 0.383 | -0.069 | -1.227** |
|  | (0.313) | (0.281) | (0.249) | (0.384) |
| DecreaseXSame religion | -0.358 | -0.233 | -0.240 | 1.163** |
|  | (0.371) | (0.327) | (0.300) | (0.433) |
| DecreaseXAlly | 0.128 | 0.332 | -0.259 | 0.635 |
|  | (0.371) | (0.325) | (0.299) | (0.442) |
| DecreaseXRival | -0.541 | 0.461 | -0.203 | 0.280 |
|  | (0.368) | (0.321) | (0.303) | (0.449) |
| DecreaseXDeveloped | -0.171 | -0.128 | -0.367 | 0.798 |
|  | (0.367) | (0.327) | (0.307) | (0.439) |
| DecreaseXUnderdeveloped | 0.018 | 0.275 | -0.329 | -0.022 |
|  | (0.367) | (0.320) | (0.304) | (0.444) |
| DecreaseXMilitary Strong | 0.033 | -0.025 | -0.235 | 0.853 |
|  | (0.367) | (0.321) | (0.299) | (0.440) |
| DecreaseXMilitary Weak | -0.980** | -0.368 | -0.273 | 1.550*** |
|  | (0.381) | (0.336) | (0.317) | (0.443) |
| IncreaseXChristian/Muslim | -0.357 | -0.472 | 0.328 | 0.357 |
|  | (0.379) | (0.329) | (0.315) | (0.436) |
| IncreaseXAlly | 0.143 | -0.191 | -0.210 | -0.223 |
|  | (0.375) | (0.328) | (0.323) | (0.446) |
| IncreaseXRival | 0.116 | 0.500 | 0.181 | 0.703 |
|  | (0.371) | (0.318) | (0.320) | (0.448) |
| IncreaseXDeveloped | -0.052 | -0.540 | -0.310 | -0.390 |
|  | (0.372) | (0.329) | (0.317) | (0.424) |
| IncreaseXUnderdeveloped | 0.252 | -0.700* | 0.159 | 0.279 |
|  | (0.369) | (0.323) | (0.358) | (0.473) |
| IncreaseXMilitary Strong | -0.050 | -0.292 | -0.168 | 0.789 |
|  | (0.372) | (0.319) | (0.320) | (0.432) |
| IncreaseXMilitary Weak | -0.460 | -0.648 | 0.247 | 0.130 |
|  | (0.378) | (0.337) | (0.325) | (0.439) |
| **Controls** | **YES** | **YES** | **YES** | **YES** |
| Constant | -1.382*** | 0.460 | 0.203 | -0.937* |
|  | (0.338) | (0.291) | (0.281) | (0.447) |
| Individual-vignette intercept | 1.037*** | 0.553*** | 0.302*** | 2.126*** |
|  | (0.121) | (0.081) | (0.070) | (0.191) |
| Number of vignettes | 5,824 | 5,824 | 6,016 | 6,041 |
| Number of individuals | 1,456 | 1,456 | 1,512 | 1,512 |

Standard errors in parentheses *** p<0.001, ** p<0.01, * p<0.05. Baseline categories “keep as is” for Policy treatment, and “no information” for signal source treatment.

**Table A-7: Policy Signal- General Pessimism Interactions: Multilevel Logit Estimates**

| **DV: Reciprocity** | **United States** | | **Turkey** | |
| --- | --- | --- | --- | --- |
|  | **UN** | **Trade** | **UN** | **Trade** |
| VARIABLES | Model-1 | Model-2 | Model-3 | Model-4 |
| Decrease | -2.806*** | -1.572*** | -0.839*** | -2.469*** |
|  | (0.232) | (0.200) | (0.146) | (0.226) |
| Increase | -2.956*** | -1.660*** | -1.835*** | -1.924*** |
|  | (0.239) | (0.199) | (0.154) | (0.223) |
| Ext. positive | -0.852** | -0.759*** | -0.324 | -0.370 |
|  | (0.265) | (0.229) | (0.208) | (0.331) |
| Somewhat positive | -0.022 | -0.475* | -0.243 | -0.281 |
|  | (0.239) | (0.198) | (0.172) | (0.276) |
| Somewhat negative | -0.759* | -0.393 | -0.217 | -0.530 |
|  | (0.297) | (0.268) | (0.181) | (0.287) |
| Ext. negative | -0.967* | -0.111 | -0.437 | -0.857* |
|  | (0.413) | (0.382) | (0.233) | (0.355) |
| DecreaseXExt. positive | 1.145*** | 1.089*** | -0.070 | 0.719* |
|  | (0.307) | (0.269) | (0.259) | (0.360) |
| DecreaseXSomewhat positive | 0.061 | 0.792*** | 0.055 | 0.487 |
|  | (0.272) | (0.232) | (0.209) | (0.297) |
| DecreaseXSomewhat negative | 1.078** | 0.861** | 0.547* | 0.670* |
|  | (0.344) | (0.314) | (0.218) | (0.310) |
| DecreaseXExt. negative | 1.573*** | 0.040 | 0.575* | 0.599 |
|  | (0.477) | (0.454) | (0.280) | (0.382) |
| IncreaseXExt. positive | 1.274*** | 0.988*** | 0.604* | 0.119 |
|  | (0.309) | (0.269) | (0.264) | (0.356) |
| IncreaseXSomewhat positive | 0.290 | 0.707** | 0.453* | 0.187 |
|  | (0.278) | (0.232) | (0.217) | (0.295) |
| IncreaseXSomewhat negative | 1.128** | 0.896** | -0.146 | 0.489 |
|  | (0.351) | (0.312) | (0.239) | (0.307) |
| IncreaseXExt. negative | 0.648 | 0.867 | 0.312 | 0.628 |
|  | (0.507) | (0.446) | (0.297) | (0.385) |
| Same Religion | -0.373** | -0.089 | 0.040 | -0.145 |
|  | (0.136) | (0.119) | (0.119) | (0.137) |
| Ally | 0.467*** | -0.056 | 0.179 | -0.170 |
|  | (0.132) | (0.118) | (0.116) | (0.138) |
| Rival | -0.262 | -0.074 | -0.028 | -0.158 |
|  | (0.136) | (0.118) | (0.119) | (0.141) |
| Developed | 0.087 | 0.011 | 0.137 | -0.844*** |
|  | (0.134) | (0.117) | (0.113) | (0.136) |
| Developing | -0.046 | -0.034 | -0.038 | -0.907*** |
|  | (0.134) | (0.117) | (0.126) | (0.147) |
| Military Strong | -0.101 | 0.049 | 0.017 | -0.271 |
|  | (0.133) | (0.118) | (0.118) | (0.139) |
| Military Weak | -0.295* | -0.025 | -0.086 | -0.523*** |
|  | (0.135) | (0.119) | (0.124) | (0.143) |
| **Demographic Controls** | **YES** | **YES** | **YES** | **YES** |
| Trust UN | 0.015 |  | -0.059 |  |
|  | (0.034) |  | (0.030) |  |
| Free Trade/ Trust_MNC |  | 0.063 |  | -0.101* |
|  |  | (0.033) |  | (0.048) |
| Constant | 1.920*** | 0.035 | 0.072 | 0.962* |
|  | (0.338) | (0.290) | (0.261) | (0.397) |
| Individual-vignette intercept | 1.053*** | 0.538*** | 0.300*** | 2.075*** |
|  | (0.122) | (0.080) | (0.070) | (0.186) |
| Observations | 5,824 | 5,824 | 6,016 | 6,041 |
| Number of groups | 1,456 | 1,456 | 1,512 | 1,512 |

*** p<0.001, ** p<0.01, * p<0.05 Baseline categories are “neither positive nor negative” for general pessimism, “keep as is” for Policy treatment, and “no information” for signal source treatment.

**A-8: Main Models with full sample (US only)**

The following models replicate the analyses on Table-2 and Appendix A-3 when respondents who fail the attention-checks in the US survey are included. Because for those respondents, data on *Trust UN* and *Free trade* are missing, these covariates are removed from the regressions.

**Table A-8a– Support for International Cooperation- Multilevel Estimates (full sample)**

| **DV: Cooperation** | **UN Experiment** | **Trade Experiment** |
| --- | --- | --- |
|  |  |  |
| VARIABLES | (1) | (2) |
| Decrease 50% | -0.428*** | -0.949*** |
|  | (0.070) | (0.090) |
| Decrease 10% | -0.186*** | -0.440*** |
|  | (0.054) | (0.085) |
| Increase 10% | 0.382*** | 0.313*** |
|  | (0.057) | (0.081) |
| Increase 50% | 0.636*** | 0.784*** |
|  | (0.069) | (0.094) |
| Economic worries | -0.449*** | 0.104* |
|  | (0.049) | (0.041) |
| Christian | 0.041 | -0.046 |
|  | (0.079) | (0.117) |
| Ally | -0.023 | -0.246* |
|  | (0.081) | (0.121) |
| Rival | 0.176* | -0.007 |
|  | (0.079) | (0.117) |
| Developed | 0.034 | -0.088 |
|  | (0.079) | (0.117) |
| Developing | 0.194* | -0.332** |
|  | (0.078) | (0.114) |
| Military Strong | 0.006 | -0.071 |
|  | (0.077) | (0.122) |
| Military Weak | 0.122 | 0.026 |
|  | (0.074) | (0.117) |
| **Demographic controls** | **YES** | **YES** |
| Constant | 7.245*** | 5.704*** |
|  | (0.286) | (0.252) |
| Individual-vignette intercept | 0.393*** | -0.205* |
|  | (0.030) | (0.081) |
| Observations | 6,056 | 6,056 |
| Number of groups | 1,514 | 1,514 |

Robust standard errors in parentheses

*** p<0.001, ** p<0.01, * p<0.05

**Table A-8b: Interaction Effects on Reciprocity- Multilevel Logit Estimates (full sample)**

| **DV: Reciprocity** | **UN Experiment** | | **Trade Experiment** | |
| --- | --- | --- | --- | --- |
|  |  | |  | |
| VARIABLES | (1) | (2) | (3) | (4) |
| Decrease | -2.097*** | -2.243*** | -0.868*** | -1.452*** |
|  | (0.094) | (0.169) | (0.082) | (0.158) |
| Increase | -2.113*** | -2.216*** | -0.968*** | -1.483*** |
|  | (0.094) | (0.169) | (0.083) | (0.157) |
| Much better | 0.026 | -0.946*** | -0.097 | -0.787** |
|  | (0.144) | (0.258) | (0.132) | (0.251) |
| Somewhat better | 0.089 | 0.737*** | 0.084 | -0.436* |
|  | (0.097) | (0.202) | (0.089) | (0.173) |
| Somewhat worse | 0.143 | -0.148 | 0.327** | -0.440 |
|  | (0.133) | (0.250) | (0.122) | (0.233) |
| Much worse | 0.200 | -1.404*** | -0.041 | -0.741* |
|  | (0.162) | (0.303) | (0.151) | (0.288) |
| DecreaseXMuch better |  | 1.188*** |  | 0.891** |
|  |  | (0.311) |  | (0.294) |
| DecreaseXSomewhat better |  | -1.140*** |  | 0.762*** |
|  |  | (0.235) |  | (0.202) |
| DecreaseXSomewhat worse |  | 0.990*** |  | 1.048*** |
|  |  | (0.289) |  | (0.273) |
| DecreaseXMuch worse |  | 2.896*** |  | 0.493 |
|  |  | (0.351) |  | (0.343) |
| IncreaseXMuch better |  | 1.308*** |  | 0.849** |
|  |  | (0.302) |  | (0.294) |
| IncreaseXSomewhat better |  | -0.440 |  | 0.542** |
|  |  | (0.232) |  | (0.202) |
| IncreaseXSomewhat worse |  | -0.441 |  | 0.874** |
|  |  | (0.308) |  | (0.273) |
| IncreaseXMuch worse |  | 1.026** |  | 1.244*** |
|  |  | (0.364) |  | (0.335) |
| Christian | -0.306* | -0.289* | -0.091 | -0.095 |
|  | (0.130) | (0.133) | (0.120) | (0.121) |
| Ally | 0.451*** | 0.448*** | -0.052 | -0.045 |
|  | (0.126) | (0.129) | (0.119) | (0.120) |
| Rival | -0.249 | -0.234 | -0.061 | -0.053 |
|  | (0.130) | (0.133) | (0.120) | (0.120) |
| Developed | 0.088 | 0.042 | -0.013 | -0.013 |
|  | (0.128) | (0.132) | (0.118) | (0.119) |
| Developing | 0.000 | -0.009 | -0.119 | -0.124 |
|  | (0.128) | (0.131) | (0.118) | (0.119) |
| Military Strong | -0.081 | -0.092 | 0.055 | 0.059 |
|  | (0.128) | (0.131) | (0.120) | (0.120) |
| Military Weak | -0.242 | -0.246 | -0.045 | -0.037 |
|  | (0.129) | (0.132) | (0.120) | (0.120) |
| **Demographic Controls** | **YES** | **YES** | **YES** | **YES** |
| Constant | 1.351*** | 1.446*** | -0.330 | 0.109 |
|  | (0.254) | (0.278) | (0.233) | (0.252) |
| Individual-vignette intercept | 0.915*** | 0.947*** | 0.755*** | 0.753*** |
|  | (0.109) | (0.114) | (0.093) | (0.093) |
| Observations | 6,056 | 6,056 | 6,056 | 6,056 |
| Number of groups | 1,514 | 1,514 | 1,514 | 1,514 |

*** p<0.001, ** p<0.01, * p<0.05 Baseline categories are “about the same” for economic worries, “keep as is” for Policy treatment, and “no information” for signal source treatment.

**Table A-9a: Interaction Effects on Strict Reciprocity- Multilevel Logit Estimates**

| **DV: Strict Reciprocity** | **United States** | | **Turkey** | |
| --- | --- | --- | --- | --- |
|  | **UN** | **Trade** | **UN** | **Trade** |
| VARIABLES | Model-1 | Model-2 | Model-3 | Model-4 |
| Decrease | -4.358*** | -2.248*** | -2.872*** | -4.687*** |
|  | (0.250) | (0.164) | (0.203) | (0.279) |
| Increase | -3.921*** | -2.269*** | -3.878*** | -4.209*** |
|  | (0.237) | (0.164) | (0.242) | (0.270) |
| Much better | -1.247*** | -0.609* | -0.588 | -1.063* |
|  | (0.351) | (0.250) | (0.381) | (0.542) |
| Somewhat better | 0.818** | -0.366* | -0.290 | -0.499 |
|  | (0.266) | (0.169) | (0.221) | (0.348) |
| Somewhat worse | -0.166 | -0.480* | -0.416* | -0.203 |
|  | (0.329) | (0.227) | (0.187) | (0.297) |
| Much worse | -1.349** | -0.417 | -0.397 | -0.568 |
|  | (0.418) | (0.299) | (0.250) | (0.364) |
| DecreaseXMuch better | 0.918* | 0.334 | -0.760 | 0.837 |
|  | (0.452) | (0.318) | (0.669) | (0.641) |
| DecreaseXSomewhat better | -0.975** | 0.537** | -0.127 | 0.816* |
|  | (0.321) | (0.208) | (0.321) | (0.394) |
| DecreaseXSomewhat worse | 1.027** | 0.925*** | 0.756** | 0.694* |
|  | (0.389) | (0.277) | (0.250) | (0.333) |
| DecreaseXMuch worse | 1.979*** | 0.445 | 0.133 | 1.274** |
|  | (0.496) | (0.369) | (0.344) | (0.407) |
| IncreaseXMuch better | 1.191** | 0.405 | 0.444 | -0.347 |
|  | (0.407) | (0.315) | (0.632) | (0.680) |
| IncreaseXSomewhat better | -0.530 | 0.507* | 1.010** | 0.415 |
|  | (0.308) | (0.208) | (0.335) | (0.388) |
| IncreaseXSomewhat worse | -0.111 | 0.901** | 0.881** | 0.575 |
|  | (0.408) | (0.278) | (0.293) | (0.326) |
| IncreaseXMuch worse | 0.653 | 0.248 | 0.904* | 0.682 |
|  | (0.530) | (0.371) | (0.372) | (0.402) |
| Same Religion | -0.438* | 0.021 | -0.053 | -0.242 |
|  | (0.185) | (0.130) | (0.170) | (0.159) |
| Ally | 0.487** | 0.133 | 0.182 | 0.003 |
|  | (0.174) | (0.129) | (0.165) | (0.161) |
| Rival | -0.315 | 0.093 | -0.179 | -0.217 |
|  | (0.182) | (0.129) | (0.172) | (0.164) |
| Developed | -0.089 | 0.182 | 0.151 | -0.673*** |
|  | (0.181) | (0.127) | (0.162) | (0.162) |
| Developing | -0.298 | -0.038 | -0.159 | -0.941*** |
|  | (0.183) | (0.130) | (0.181) | (0.180) |
| Military Strong | -0.264 | 0.104 | -0.021 | -0.312 |
|  | (0.179) | (0.129) | (0.169) | (0.163) |
| Military Weak | -0.437* | 0.091 | -0.248 | -0.620*** |
|  | (0.182) | (0.130) | (0.178) | (0.171) |
| Demographic Controls | YES | YES | YES | YES |
| Trust UN | 0.239*** |  | 0.108* |  |
|  | (0.056) |  | (0.047) |  |
| Free Trade/Trust_MNC |  | 0.044 |  | -0.001 |
|  |  | (0.035) |  | (0.060) |
| Constant | 1.344** | -0.105 | 0.306 | 0.282 |
|  | (0.448) | (0.278) | (0.377) | (0.476) |
| Individual-vignette intercept | 2.838*** | 0.464*** | 0.992*** | 3.510*** |
|  | (0.298) | (0.085) | (0.161) | (0.317) |
| Observations | 5,824 | 5,824 | 6,016 | 6,041 |
| Number of groups | 1,456 | 1,456 | 1,512 | 1,512 |

*** p<0.001, ** p<0.01, * p<0.05 Baseline categories are “about the same” for economic worries, “keep as is” for Policy treatment, and “no information” for signal source treatment.

**Table A-9b: Test of Mean Differences across values of Economic Worries and Policy Treatment –Strict reciprocity**

| ***United States-UN*** | | | |
| --- | --- | --- | --- |
| ***Treatment*** | ***Difference*** | ***standard error*** | ***p-value*** |
| Much worse X increase (N=178) vs Much better X increase (N=293) | -0.107 | 0.034 | **0.002** |
| Much worse X decrease (N=166) vs Much better X decrease (N=240) | 0.052 | 0.035 | 0.136 |
| Somewhat worse X increase (N=311) vs Somewhat better X increase (N=996) | -0.075 | 0.026 | **0.003** |
| Somewhat worse X decrease (N=326) vs Somewhat better X decrease (N=1030) | 0.081 | 0.022 | **0.000** |
|  |  |  |  |
| ***United States-Trade*** | | | |
| ***Treatment*** | ***Difference*** | ***standard error*** | ***p-value*** |
| Much worse X increase (N=168) vs Much better X increase (N=269) | 0.002 | 0.042 | 0.968 |
| Much worse X decrease (N=170) vs Much better X decrease (N=270) | 0.063 | 0.039 | 0.107 |
| Somewhat worse X increase (N=302) vs Somewhat better X increase (N=987) | 0.057 | 0.029 | **0.050** |
| Somewhat worse X decrease (N=331) vs Somewhat better X decrease (N=1028) | 0.045 | 0.028 | 0.115 |
|  |  |  |  |
| ***Turkey-UN*** | | | |
| ***Treatment*** | ***Difference*** | ***standard error*** | ***p-value*** |
| Much worse X increase (N=354) vs Much better X increase (N=110) | -0.033 | 0.028 | 0.231 |
| Much worse X decrease (N=347) vs Much better X decrease (N=98) | 0.049 | 0.031 | 0.116 |
| Somewhat worse X increase (N=908) vs Somewhat better X increase (N=420) | -0.021 | 0.016 | 0.182 |
| Somewhat worse X decrease (N=876) vs Somewhat better X decrease (N=401) | 0.065 | 0.020 | **0.001** |
|  |  |  |  |
| ***Turkey-Trade*** | | | |
| ***Treatment*** | ***Difference*** | ***standard error*** | ***p-value*** |
| Much worse X increase (N=342) vs Much better X increase (N=97) | 0.156 | 0.049 | **0.002** |
| Much worse X decrease (N=330) vs Much better X decrease (N=106) | 0.159 | 0.048 | **0.001** |
| Somewhat worse X increase (N=897) vs Somewhat better X increase (N=434) | 0.063 | 0.026 | **0.017** |
| Somewhat worse X decrease (N=900) vs Somewhat better X decrease (N=413) | 0.000 | 0.026 | 0.984 |

Note: The t statistic is obtained from a *t* test comparing selected treatment groups with symmetrically opposite economic expectations. *p*-values in bold denote statistical significance at 0.05 level.

**Table B-1 Frequency Distributions between Policy Signal and Cooperation**

| a- US- UN Experiment | | | |  |  |  |  |  |  |  |  | |
| --- | --- | --- | --- | --- | --- | --- | --- | --- | --- | --- | --- | --- |
| Cooperation→ | Dec. >50% | Dec. 50% | Dec 10-9% | Dec. 10% | Dec. 0-9% | Keep same | Inc. 0-9% | Inc. 10% | Inc. 11-49% | Inc. 50% | Inc. > 50% | |
| Policy Treatment↓ | |  |  |  |  |  |  |  |  |  |  | |
| Decrease 50% | 97 | 165 | 75 | 32 | 29 | 668 | 8 | 58 | 50 | 33 | 12 | |
|  | 7.91 | 13.45 | 6.11 | 2.61 | 2.36 | 54.44 | 0.65 | 4.73 | 4.07 | 2.69 | 0.98 | |
|  |  |  |  |  |  |  |  |  |  |  |  | |
| Decrease 10% | 92 | 24 | 37 | 170 | 27 | 751 | 21 | 62 | 23 | 6 | 9 | |
|  | 7.53 | 1.96 | 3.03 | 13.91 | 2.21 | 61.46 | 1.72 | 5.07 | 1.88 | 0.49 | 0.74 | |
|  |  |  |  |  |  |  |  |  |  |  |  | |
| Keep as is | 87 | 18 | 47 | 37 | 15 | 901 | 13 | 50 | 22 | 8 | 12 | |
|  | 7.19 | 1.49 | 3.88 | 3.06 | 1.24 | 74.46 | 1.07 | 4.13 | 1.82 | 0.66 | 0.99 | |
|  |  |  |  |  |  |  |  |  |  |  |  | |
| Increase 10% | 83 | 23 | 39 | 37 | 10 | 675 | 29 | 267 | 47 | 17 | 4 | |
|  | 6.74 | 1.87 | 3.17 | 3.01 | 0.81 | 54.83 | 2.36 | 21.69 | 3.82 | 1.38 | 0.32 | |
|  |  |  |  |  |  |  |  |  |  |  |  | |
| Increase 50% | 73 | 32 | 45 | 40 | 11 | 673 | 16 | 63 | 100 | 164 | 25 | |
|  | 5.88 | 2.58 | 3.62 | 3.22 | 0.89 | 54.19 | 1.29 | 5.07 | 8.05 | 13.2 | 2.01 | |
|  |  |  |  |  |  |  |  |  |  |  |  | |
| Total | 432 | 262 | 243 | 316 | 92 | 3,668 | 87 | 500 | 242 | 228 | 62 | |
|  | 7.05 | 4.27 | 3.96 | 5.15 | 1.5 | 59.82 | 1.42 | 8.15 | 3.95 | 3.72 | 1.01 | |
| The two-way table of frequency between Policy treatment(5 cat.-left) and Cooperation (11-level, top). For each policy treatment row, the upper cells report the frequency count, and the lower ones, the frequency percentage. Highlighted cells denote responses in line with the signal direction and constitutes our second DV, *reciprocity.* The boldly bordered cells denote responses that match both the direction and degree of the treatment signal, i.e. *strict reciprocity*. | | | | | | | | | | | |  |
|  |  |  |  |  |  |  |  |  |  |  |  |  |

| b- US- Trade Experiment | | | |  |  |  |  |  |  |  |  | |
| --- | --- | --- | --- | --- | --- | --- | --- | --- | --- | --- | --- | --- |
| Cooperation→ | Dec. >50% | Dec. 50% | Dec 10-9% | Dec. 10% | Dec. 0-9% | Keep same | Inc. 0-9% | Inc. 10% | Inc. 11-49% | Inc. 50% | Inc. > 50% | |
| Policy Treatment↓ | |  |  |  |  |  |  |  |  |  |  | |
| Decrease 50% | 23 | 275 | 141 | 99 | 27 | 460 | 7 | 71 | 43 | 66 | 28 | |
|  | 1.85 | 22.18 | 11.37 | 7.98 | 2.18 | 37.1 | 0.56 | 5.73 | 3.47 | 5.32 | 2.26 | |
|  |  |  |  |  |  |  |  |  |  |  |  | |
| Decrease 10% | 20 | 59 | 52 | 348 | 61 | 450 | 12 | 97 | 35 | 60 | 34 | |
|  | 1.63 | 4.8 | 4.23 | 28.34 | 4.97 | 36.64 | 0.98 | 7.9 | 2.85 | 4.89 | 2.77 | |
|  |  |  |  |  |  |  |  |  |  |  |  | |
| Keep as is | 15 | 50 | 39 | 82 | 24 | 782 | 12 | 86 | 41 | 58 | 36 | |
|  | 1.22 | 4.08 | 3.18 | 6.69 | 1.96 | 63.84 | 0.98 | 7.02 | 3.35 | 4.73 | 2.94 | |
|  |  |  |  |  |  |  |  |  |  |  |  | |
| Increase 10% | 12 | 60 | 43 | 101 | 26 | 446 | 20 | 331 | 72 | 67 | 29 | |
|  | 0.99 | 4.97 | 3.56 | 8.37 | 2.15 | 36.95 | 1.66 | 27.42 | 5.97 | 5.55 | 2.4 | |
|  |  |  |  |  |  |  |  |  |  |  |  | |
| Increase 50% | 7 | 62 | 59 | 97 | 16 | 470 | 8 | 83 | 66 | 276 | 88 | |
|  | 0.57 | 5.03 | 4.79 | 7.87 | 1.3 | 38.15 | 0.65 | 6.74 | 5.36 | 22.4 | 7.14 | |
|  |  |  |  |  |  |  |  |  |  |  |  | |
| Total | 77 | 506 | 334 | 727 | 154 | 2,608 | 59 | 668 | 257 | 527 | 215 | |
|  | 1.26 | 8.25 | 5.45 | 11.86 | 2.51 | 42.53 | 0.96 | 10.89 | 4.19 | 8.59 | 3.51 | |
| The two-way table of frequency between Policy treatment(5 cat.-left) and Cooperation (11-level, top). For each policy treatment row, the upper cells report the frequency count, and the lower ones, the frequency percentage. Highlighted cells denote responses in line with the signal direction and constitutes our second DV, *reciprocity.* The boldly bordered cells denote responses that match both the direction and degree of the treatment signal, i.e. *strict reciprocity*. | | | | | | | | | | | |  |
|  |  |  |  |  |  |  |  |  |  |  |  |  |

| c- Turkey- UN Experiment | | | |  |  |  |  |  |  |  |  | |
| --- | --- | --- | --- | --- | --- | --- | --- | --- | --- | --- | --- | --- |
| Cooperation→ | Dec. >50% | Dec. 50% | Dec 10-9% | Dec. 10% | Dec. 0-9% | Keep same | Inc. 0-9% | Inc. 10% | Inc. 11-49% | Inc. 50% | Inc. > 50% | |
| Policy Treatment↓ | |  |  |  |  |  |  |  |  |  |  | |
| Decrease 50% | 128 | 142 | 76 | 81 | 45 | 560 | 29 | 52 | 30 | 24 | 19 | |
|  | 10.79 | 11.97 | 6.41 | 6.83 | 3.79 | 47.22 | 2.45 | 4.38 | 2.53 | 2.02 | 1.6 | |
|  |  |  |  |  |  |  |  |  |  |  |  | |
| Decrease 10% | 95 | 63 | 74 | 127 | 85 | 549 | 46 | 58 | 31 | 13 | 20 | |
|  | 8.18 | 5.43 | 6.37 | 10.94 | 7.32 | 47.29 | 3.96 | 5 | 2.67 | 1.12 | 1.72 | |
|  |  |  |  |  |  |  |  |  |  |  |  | |
| Keep as is | 83 | 76 | 64 | 79 | 43 | 643 | 46 | 69 | 40 | 25 | 24 | |
|  | 6.96 | 6.38 | 5.37 | 6.63 | 3.61 | 53.94 | 3.86 | 5.79 | 3.36 | 2.1 | 2.01 | |
|  |  |  |  |  |  |  |  |  |  |  |  | |
| Increase 10% | 94 | 71 | 45 | 64 | 57 | 628 | 46 | 107 | 41 | 26 | 17 | |
|  | 7.86 | 5.94 | 3.76 | 5.35 | 4.77 | 52.51 | 3.85 | 8.95 | 3.43 | 2.17 | 1.42 | |
|  |  |  |  |  |  |  |  |  |  |  |  | |
| Increase 50% | 87 | 69 | 55 | 57 | 39 | 706 | 45 | 89 | 43 | 67 | 30 | |
|  | 6.76 | 5.36 | 4.27 | 4.43 | 3.03 | 54.86 | 3.5 | 6.92 | 3.34 | 5.21 | 2.33 | |
|  |  |  |  |  |  |  |  |  |  |  |  | |
| Total | 487 | 421 | 314 | 408 | 269 | 3,086 | 212 | 375 | 185 | 155 | 110 | |
|  | 8.09 | 6.99 | 5.21 | 6.78 | 4.47 | 51.25 | 3.52 | 6.23 | 3.07 | 2.57 | 1.83 | |
| The two-way table of frequency between Policy treatment(5 cat.-left) and Cooperation (11-level, top). For each policy treatment row, the upper cells report the frequency count, and the lower ones, the frequency percentage. Highlighted cells denote responses in line with the signal direction and constitutes our second DV, *reciprocity.* The boldly bordered cells denote responses that match both the direction and degree of the treatment signal, i.e. *strict reciprocity*. | | | | | | | | | | | |  |
|  |  |  |  |  |  |  |  |  |  |  |  |  |

| d- Turkey- Trade Experiment | | | | |  |  |  |  |  |  |  |
| --- | --- | --- | --- | --- | --- | --- | --- | --- | --- | --- | --- |
| Cooperation→ | Dec. >50% | Dec. 50% | Dec 10-9% | Dec. 10% | Dec. 0-9% | Keep same | Inc. 0-9% | Inc. 10% | Inc. 11-49% | Inc. 50% | Inc. > 50% |
| Policy Treatment↓ | |  |  |  |  |  |  |  |  |  |  |
| Decrease 50% | 32 | 220 | 121 | 135 | 77 | 444 | 5 | 17 | 22 | 25 | 24 |
|  | 2.85 | 19.61 | 10.78 | 12.03 | 6.86 | 39.57 | 0.45 | 1.52 | 1.96 | 2.23 | 2.14 |
|  |  |  |  |  |  |  |  |  |  |  |  |
| Decrease 10% | 22 | 25 | 65 | 366 | 129 | 494 | 12 | 86 | 42 | 17 | 26 |
|  | 1.71 | 1.95 | 5.06 | 28.5 | 10.05 | 38.47 | 0.93 | 6.7 | 3.27 | 1.32 | 2.02 |
|  |  |  |  |  |  |  |  |  |  |  |  |
| Keep as is | 12 | 20 | 20 | 53 | 42 | 959 | 8 | 24 | 20 | 16 | 22 |
|  | 1 | 1.67 | 1.67 | 4.43 | 3.51 | 80.18 | 0.67 | 2.01 | 1.67 | 1.34 | 1.84 |
|  |  |  |  |  |  |  |  |  |  |  |  |
| Increase 10% | 14 | 23 | 31 | 96 | 59 | 407 | 27 | 340 | 155 | 48 | 39 |
|  | 1.13 | 1.86 | 2.5 | 7.75 | 4.76 | 32.85 | 2.18 | 27.44 | 12.51 | 3.87 | 3.15 |
|  |  |  |  |  |  |  |  |  |  |  |  |
| Increase 50% | 21 | 25 | 21 | 44 | 26 | 345 | 15 | 70 | 115 | 324 | 194 |
|  | 1.75 | 2.08 | 1.75 | 3.67 | 2.17 | 28.75 | 1.25 | 5.83 | 9.58 | 27 | 16.17 |
|  |  |  |  |  |  |  |  |  |  |  |  |
| Total | 101 | 313 | 258 | 694 | 333 | 2,649 | 67 | 537 | 354 | 430 | 305 |
|  | 1.67 | 5.18 | 4.27 | 11.49 | 5.51 | 43.85 | 1.11 | 8.89 | 5.86 | 7.12 | 5.05 |
| The two-way table of frequency between Policy treatment(5 cat.-left) and Cooperation (11-level, top). For each policy treatment row, the upper cells report the frequency count, and the lower ones, the frequency percentage. Highlighted cells denote responses in line with the signal direction and constitutes our second DV, *reciprocity.* The boldly bordered cells denote responses that match both the direction and degree of the treatment signal, i.e. *strict reciprocity*. | | | | | | | | | | | |
|  |  |  |  |  |  |  |  |  |  |  |  |

**Table B-2: Country-level Differences in Treatment Effects**

| **DV:** | **Cooperation** | | **Reciprocity** | |  |
| --- | --- | --- | --- | --- | --- |
|  | **UN** | **Trade** | **UN** | **Trade** |  |
| VARIABLES | (1) | (2) | (3) | (4) |  |
| Decrease | -0.298*** | 0.895*** | -0.671*** | -1.817*** |  |
|  | (0.048) | (0.059) | (0.111) | (0.125) |  |
| Increase | 0.218*** | -1.444*** | -1.705*** | -1.518*** |  |
|  | (0.049) | (0.065) | (0.112) | (0.119) |  |
| USAXDecrease | -0.003 | -0.247** | -1.491*** | 0.855*** |  |
|  | (0.068) | (0.093) | (0.167) | (0.157) |  |
| USAXIncrease | 0.353*** | 0.867*** | -0.499** | 0.473** |  |
|  | (0.069) | (0.097) | (0.155) | (0.153) |  |
| USA | -0.262 | 0.127 | 0.775** | -1.184*** |  |
|  | (0.323) | (0.244) | (0.277) | (0.304) |  |
| Economic worries | -0.161*** | -0.020 | 0.029 | 0.086* |  |
|  | (0.048) | (0.035) | (0.033) | (0.044) |  |
| USAXEconomic worries | -0.047 | -0.077 | -0.003 | -0.047 |  |
|  | (0.068) | (0.053) | (0.052) | (0.056) |  |
| Trust UN | 0.477*** |  | -0.070* |  |  |
|  | (0.048) |  | (0.031) |  |  |
| USAXTrust UN | 0.130* |  | 0.119** |  |  |
|  | (0.062) |  | (0.045) |  |  |
| Same Religion | 0.127 | 0.142 | 0.032 | -0.138 |  |
|  | (0.077) | (0.090) | (0.120) | (0.115) |  |
| Ally | -0.149* | 0.287** | 0.174 | -0.177 |  |
|  | (0.068) | (0.093) | (0.114) | (0.114) |  |
| Rival | -0.042 | -0.267** | -0.029 | -0.156 |  |
|  | (0.069) | (0.092) | (0.119) | (0.116) |  |
| Developed | -0.156* | 0.397*** | 0.124 | -0.757*** |  |
|  | (0.073) | (0.091) | (0.115) | (0.115) |  |
| Developing | 0.084 | -0.261* | -0.059 | -0.830*** |  |
|  | (0.078) | (0.101) | (0.129) | (0.133) |  |
| Military Strong | -0.109 | -0.060 | 0.009 | -0.267* |  |
|  | (0.070) | (0.085) | (0.119) | (0.119) |  |
| Military Weak | 0.071 | 0.279** | -0.122 | -0.472*** |  |
|  | (0.070) | (0.090) | (0.132) | (0.126) |  |
| USAXSame Religion | -0.126 | -0.158 | -0.334 | -0.072 |  |
|  | (0.104) | (0.146) | (0.173) | (0.171) |  |
| USAXAlly | 0.085 | -0.081 | 0.279 | -0.142 |  |
|  | (0.102) | (0.151) | (0.167) | (0.168) |  |
| USAXRival | 0.168 | 0.285 | -0.217 | 0.053 |  |
|  | (0.101) | (0.149) | (0.171) | (0.171) |  |
| USAXDeveloped | 0.126 | -0.321* | -0.006 | 0.789*** |  |
|  | (0.104) | (0.146) | (0.167) | (0.174) |  |
| USAXDeveloping | 0.045 | 0.529*** | 0.055 | 0.863*** |  |
|  | (0.105) | (0.151) | (0.178) | (0.183) |  |
| USAXMilitary Strong | 0.087 | 0.107 | -0.087 | 0.238 |  |
|  | (0.102) | (0.146) | (0.172) | (0.173) |  |
| USAXMilitary Weak | -0.008 | -0.317* | -0.145 | 0.375* |  |
|  | (0.099) | (0.146) | (0.180) | (0.177) |  |
| Constant | 4.592*** | 5.967*** | 0.260 | 2.040*** |  |
|  | (0.244) | (0.182) | (0.184) | (0.249) |  |
| Individual-vignette intercept | 0.455*** | -0.022 | 0.646*** | 1.221*** |  |
|  | (0.018) | (0.040) | (0.070) | (0.094) |  |
| Observations | 0.226*** | 0.667*** | 12,144 | 12,169 |  |
| Number of groups | (0.016) | (0.010) | 3,044 | 3,044 |  |

Robust standard errors in parentheses

*** p<0.001, ** p<0.01, * p<0.05
